# A Method for Predicting Tool Wear Under Different Milling Conditions by Integrating Adaptive VMD with a Two-Stage Attention Mechanism

**DOI:** 10.3390/s26216829

**Published:** 2026-10-29

**Authors:** Changjian Wen, Xiaoyu Li, Haipeng Su, Zhipeng Sun, Jian Miao, Juan Lu

**Affiliations:** Guangxi Key Laboratory of Ocean Engineering Equipment and Technology, Beibu Gulf University, Qinzhou 535011, China; 2402010120@stu.bbgu.edu.cn (C.W.); 2402010107@stu.bbgu.edu.cn (X.L.); 2402010112@stu.bbgu.edu.cn (H.S.); 15769479929@163.com (Z.S.); miaojian@bbgu.edu.cn (J.M.)

**Keywords:** tool condition monitoring, adaptive variational mode decomposition, multi-sensor data fusion, attention mechanism, tool wear prediction

## Abstract

Reliable tool condition monitoring under high-noise and different milling conditions requires robust signal preprocessing and adaptive feature extraction. This study proposes a tool wear prediction framework that integrates Adaptive Variational Mode Decomposition (AVMD) with a two-stage Convolutional Block Attention Module (CBAM). AVMD uses signal spectral features to guide a directed search for decomposition parameters, reducing reliance on manually specified settings. The reference architecture processes reconstructed multi-sensor signals through multiscale convolution and a DenseNet–BiLSTM network, with two CBAM stages for feature recalibration. The first CBAM follows multiscale convolution and precedes DenseNet, while the second follows BiLSTM. Using the selected configurations, within-tool evaluations achieve R^2^ values of 0.9399–0.9573 on TC18 titanium-alloy milling data and 0.9907–0.9929 on the public PHM 2010 dataset. Exploratory cross-tool evaluations on PHM 2010 yield R^2^ values of 0.8954–0.9599 under fixed cutting conditions. t-distributed stochastic neighbor embedding and Shapley additive explanations are used to visualize feature representations and assess feature contributions to model predictions. The results demonstrate the potential of the framework for multi-sensor tool wear estimation in the evaluated machining scenarios.

## 1. Introduction

In modern smart manufacturing, CNC machine tools constitute essential production equipment. Tool wear occurs inevitably during machining and accumulates over time. This process affects machining accuracy, surface quality, and production efficiency. The effective service life of cutting tools is typically 50–80% of the theoretical value, and unplanned downtime accounts for 7–20% of total downtime [1,2]. This situation results in reduced workpiece quality and a 5–15% increase in unit cost. In addition, incorrect assessment of tool wear conditions may cause tool breakage, equipment damage, or a production line shutdown, leading to substantial economic losses [3,4]. Therefore, online monitoring of tool wear is an important approach for maintaining machining quality, controlling production cost, and improving the reliability of smart manufacturing systems [5].

Advancements in sensor and computer technologies have facilitated the real-time acquisition of signals from multiple sensor sources, including cutting force, vibration, and acoustic emission. The integration of these signals with deep learning enables online prediction of tool wear and supports intelligent decision-making, which constitutes a critical element in constructing highly reliable intelligent manufacturing systems. This technical methodology is classified as the indirect monitoring model [6] and currently represents the principal approach for tool wear monitoring.

Indirect tool wear monitoring generally comprises three principal stages: signal preprocessing, feature extraction, and prediction. In manufacturing environments that exhibit elevated noise levels and diverse operating conditions, raw sensor signals frequently contain substantial noise interference. Such interference directly influences the accuracy of subsequent feature extraction and model performance. As a result, signal preprocessing is essential for establishing reliable relationships in wear mapping. Signal preprocessing commonly includes downsampling, filtering, noise reduction, and signal decomposition. These steps enhance signal quality and provide a solid foundation for the precise extraction of wear features. Within these procedures, signal decomposition serves as a crucial adaptive analysis technique in preprocessing [7], allowing for the decomposition of complex, non-stationary signals into a series of eigenmodes [8,9]. Signal decomposition represents complex signals as mode components with different frequency characteristics. Selected IMF components can then be used for signal reconstruction according to predefined selection criteria [10,11,12,13,14,15], providing inputs for subsequent feature extraction and prediction [16]. Empirical Mode Decomposition (EMD) represents a widely adopted signal decomposition technique [17]. Although EMD offers adaptive decomposition capabilities, it remains vulnerable to mode aliasing and end-point effects, which restrict its utility under complex operating conditions. To address issues such as envelope error accumulation, modal confusion, and end-point effects inherent in the EMD method, Dragomiretskiy et al. [18] introduced Variational Mode Decomposition (VMD). This approach possesses a robust mathematical foundation and eliminates the need to define wavelet bases as required in wavelet decomposition. By solving a constrained variational problem, VMD decomposes a signal into a prescribed number of band-limited modes, providing a framework for analyzing non-stationary sensor signals. Consequently, VMD has been applied to machining-signal analysis and related condition-monitoring tasks. For instance, Zhao et al. [19] combined optimized VMD and wavelet denoising with an SE–DCNN–GRU model for chatter monitoring in thin-walled titanium-alloy polish grinding, reporting recognition accuracy of up to 98.8% and above 95% at a noise level of −5 dB. Wang et al. [20] combined VMD with a back-propagation neural network for spindle-power modeling in tool wear monitoring, reporting an average power-prediction error of 1.1256%. Yuan et al. [21] applied VMD to decompose non-stationary current signals, extract frequency band energy, and integrate time-frequency features, achieving a wear identification accuracy of 95.19% under multiple operating conditions using a random forest. Gao et al. [22] used the Rime Ice Optimization Algorithm to optimize VMD processing of load sequences, enabling the prediction model to achieve the lowest error across various scenarios. Sefene et al. integrated VMD with machine-learning models to predict CO_2_ emissions during multi-diamond wire sawing of 4H-SiC [23]. Their study provides a related application of decomposition-based prediction in machining, with a prediction target distinct from tool wear.

Nevertheless, the effectiveness of VMD in signal decomposition is highly contingent upon its internal parameter settings. To reduce dependence on expert experience, researchers have employed heuristic algorithms to achieve adaptive optimization of internal parameters. Gao et al. [24] sought to minimize envelope entropy and used Differential Evolution (DE) to adaptively optimize VMD parameters, thereby effectively extracting weak wear signals from highly noisy vibration data. In a related machinery-diagnosis application, Wang et al. [25] used a genetic algorithm to optimize the mode number and penalty factor of VMD and combined the reconstructed signals with Lempel–Ziv complexity for quantitative rolling-bearing fault diagnosis under varying operating conditions. However, the computational cost and variability of these methods depend on their objective functions, search bounds, evaluation budgets, and stopping criteria. This motivates the investigation of spectral-feature-guided parameter selection, with its search efficiency, parameter stability, and downstream prediction performance assessed under explicitly reported experimental settings.

In tool condition monitoring, feature extraction is required to obtain sensitive information related to tool degradation from preprocessed sensor signals. Traditional feature extraction methods rely on the manual design and selection of time-domain, frequency-domain, and time–frequency-domain features, and depend strongly on prior knowledge. In recent years, deep learning techniques have been widely applied in tool condition monitoring due to their ability to learn discriminative features directly from raw data. Convolutional neural networks (CNNs) can extract local spatial features effectively [26,27,28]. However, standard CNNs operate with fixed receptive fields and typically capture single-scale features, which limits their ability to represent global wear variations [29,30]. In addition, CNNs show limited capability in modeling temporal dependencies in long-sequence signals. Recurrent neural networks (RNNs) and their variants, such as LSTM and GRU, are effective in modeling temporal dynamics [31,32], but their capacity to represent spatial structural information remains limited. To address these limitations, hybrid deep learning models have been developed to integrate spatial feature extraction and temporal modeling. Multi-Scale Convolution (MSC) extracts features using convolutional branches with different effective receptive fields. Such receptive fields can be obtained through different kernel sizes or dilation rates, allowing signal patterns at multiple scales to be represented. As a result, MSC has been applied in fault diagnosis and prediction tasks. Wang et al. [33] proposed a Multi-Scale Convolution Attention Network (MSCAN), which achieved high-accuracy remaining useful life prediction on a full-lifecycle milling dataset through multi-scale feature fusion and channel attention mechanisms. Chen et al. [34] combined a multi-scale CNN with an LSTM for bearing fault diagnosis using raw vibration signals, achieving an average classification accuracy of 98.46%. For tool wear estimation, multiscale feature extraction needs to be considered together with feature reuse and sequence modeling. Their respective contributions should therefore be assessed within a common prediction framework.

DenseNet is widely used as an effective spatial feature extractor due to its dense connection mechanism, which facilitates gradient propagation and feature reuse. Kuo et al. [35] applied DenseNet to extract multi-level reusable features from force and vibration signals and achieved high-accuracy tool wear prediction. Chehrehzad et al. [36] developed a stacked architecture by connecting multiple DenseNet modules in series, which increased network depth and receptive field and improved the capacity for multi-scale feature extraction.

BiLSTM has also been applied in tool wear prediction because it can capture dependencies in both directions within an input sequence. In the present framework, this sequence comprises signal samples within a cutting pass, allowing the network to represent temporal patterns in the acquired sensor signals. Pour et al. [37] combined BiLSTM with a Temporal Convolutional Network (TCN) to extract temporal features and achieved multi-time-window remaining useful life prediction, with a 5.5% reduction in benchmark RMSE. Although the combination of DenseNet and BiLSTM enables joint extraction of spatial and temporal features, several limitations remain. The dense connection mechanism in DenseNet introduces redundant features, which reduces feature efficiency. In addition, BiLSTM requires substantial computational resources and lacks an adaptive mechanism for weighting multi-sensor inputs. These factors limit generalization performance in complex and noisy environments and constrain further improvement in model accuracy. To address these issues, it is necessary to introduce mechanisms that can selectively enhance informative features while suppressing redundancy. Attention mechanisms provide an effective solution by assigning adaptive weights to feature channels and spatial regions, thereby improving feature representation. Hou et al. [38] combined domain-adversarial adaptation with a multi-scale temporal network incorporating channel attention and improved tool wear prediction accuracy. Huang et al. [39] developed a multi-scale CNN with an attention fusion structure and demonstrated that attention mechanisms can enhance the extraction of degradation-related features. Liang et al. [40] incorporated CBAM into an attention-augmented separable residual network for bearing fault diagnosis, improving diagnostic accuracy while reducing training time on both benchmark and engineering datasets. This result indicates that attention mechanisms can focus on critical fault-related regions and strengthen feature extraction. Related studies on bearing prognostics have also explored graph-based degradation representations. Jin et al. [41] proposed a physics-informed hypergraph wavelet attention network for multiscale bearing remaining useful life prediction. Jin et al. [42] developed a dual correlation graph knowledge distillation network that combines dynamic graph learning with multilevel spatiotemporal knowledge distillation for bearing prognostics. Existing multisensor and attention-based methods provide a foundation for combining feature extraction, sequence modeling, and adaptive feature weighting [28,29,38,39]. Building on these approaches, the present study investigates the integration of spectral-feature-guided VMD parameter selection with CBAM at two distinct processing stages of an MSC–DenseNet–BiLSTM framework. The contributions of the preprocessing strategy and individual network components are examined through input comparisons and component ablation studies.

Based on the above analysis, two issues motivate this study: the dependence of VMD preprocessing on parameter selection and the coordination of multiscale feature extraction, feature reuse, and sequence modeling. This paper develops a tool wear prediction framework integrating Adaptive Variational Mode Decomposition (AVMD) with an MSC–DenseNet–BiLSTM network and two-stage attention.

AVMD uses signal spectral characteristics to define candidate settings for the mode number *K*, penalty factor *α*, and update parameter *τ*. A coarse-to-fine search evaluates these candidates using reconstruction-based screening and ranking, as described in Section 2.2.1 and Algorithm 1. The strategy is evaluated against PSO-based, DE-based, and entropy-based VMD optimization in terms of search cost, parameter variability, and downstream wear-prediction performance.
**Algorithm 1:** Adaptive VMD Parameter Optimization**Input:** Signal set *S* for one sensor channel; candidate-generation rules in Step 1; iteration limit MaxIter = 500. **Output:** Selected parameters *θ*_opt_ = (*K*_opt_, *α*_opt_, *τ*_opt_), decomposed IMFs and reconstructed signals. 1   **Function** AdaptiveVMD(*S*) 2     X ← truncate or edge-pad *S* to 5000 samples; remove means and normalize by maximum absolute amplitude. 3     R ← representative signals selected by similarity clustering, using the threshold 0.15. 4     Initialize channel record dictionary *D* ← empty. 5     for each representative signal *x* in *R* do 6       C ← spectral-feature-guided candidate combinations (*K*, *α*, *τ*), generated according to Step 1. 7       F ← Evaluate(*x*, *θ*, 0.01, MaxIter) for all *θ* in *C*, evaluated in parallel. 8       Discard failed evaluations and records with nonfinite metrics or NMSE ≥ 100,000. 9       Q ← records in *F* satisfying SNR ≥ 8 dB and RMSE ≤ 0.1. 10        if *Q* is empty then *Q* ← the five best available records in *F*, ranked by SNR↓, RMSE↑, NMSE↑. 11        Sort *Q* by SNR↓, RMSE↑, NMSE↑; retain its first 70%, rounded down, with at least two when available. 12        Initialize *v* = *s* = *b* = 0; previous NMSE unset; best SNR = 0, best NMSE = ∞, best *q* = 0. 13        for each retained parameter combination *θ* in *Q* do 14          E ← Evaluate(*x*, *θ*, 10^−7^, MaxIter); skip if evaluation fails or NMSE ≥ 100,000. 15          Read SNR, RMSE, NMSE and *q* from *E*; store/replace *D*[*θ*] ← *E*; *v* ← *v* + 1; updated ← false. 16          if *E* passes both thresholds and improves the local best by SNR↓, NMSE↑ then updated ← true. 17          if *E* fails either threshold and *q* > best *q* then updated ← true. 18          if updated then copy current SNR, NMSE and *q* to the local best; reset *s* = *b* = 0. 19          if NMSE ≤ 0.0015 then *s* ← *s* + 1; *b* ← *b* + 1 20          else if a previous NMSE is available then 21           Δ ← absolute difference between current and previous NMSE. 22           if Δ ≤ 0.0015 or Δ/(previous NMSE + 10^−8^) < 0.0015 then *s* ← *s* + 1; else *s* ← 0. 23          end if 24          previous NMSE ← current NMSE. 25          if *v* ≥ 15 and (*s* ≥ 6 or *b* ≥ 4) then break. 26       end for 27     end for 28     V ← records in *D* satisfying SNR ≥ 8 dB and RMSE ≤ 0.1; if *V* is empty then *V* ← all valid records in *D*. 29     if *V* is empty then return a channel-calibration failure. 30     *θ*_opt_ ← highest-ranked parameter combination in *V*, ordered by SNR↓, RMSE↑, NMSE↑. 31     For each *x* in *X*, call Evaluate(*x*, *θ*_opt_, 10^−7^, MaxIter) and retain its IMFs and reconstruction. 32     return *θ*_opt_, decomposed IMFs and reconstructed signals. 33   **End Function** 34   **Function** Evaluate(*x*, *θ* = (*K*, *α*, *τ*), *ε*, MaxIter) 35     Mean-center *x*, apply mirror extension and calculate its one-sided Fourier representation. 36     Initialize modal spectra and multiplier to zero; distribute *K* center frequencies uniformly; set *n* = 0. 37     repeat 38       Sequentially update each modal spectrum using Equation (3) and its center frequency using Equation (4). 39       ${\hat{\lambda }}^{n+1}(\omega )\leftarrow {\hat{\lambda }}^{n}(\omega )+\tau [\sum_{k=1}^{K}{\hat{u}}_{k}^{n+1}(\omega )-\hat{f}(\omega )]$ 40       *n* ← *n* + 1; calculate the convergence difference using Equation (5). 41     until the convergence difference ≤ *ε* or the prescribed iteration limit is reached. 42     Recover the time-domain IMFs and remove the mirror extension. 43     Keep IMFs with absolute Pearson correlation ≥ 0.1 to *x*; if fewer than two qualify, keep the top two. 44     r ← sum of retained IMFs; *q* ← number of retained IMFs divided by *K*. 45     Calculate reconstruction SNR, RMSE and NMSE according to their definitions in Section 2.2.1. 46     return all IMFs, reconstruction *r*, SNR, RMSE, NMSE and retained-mode fraction *q*. 47   **End Function**

The reference prediction architecture combines MSC, DenseNet, BiLSTM, and two CBAM stages. The first CBAM is placed after MSC and before DenseNet to recalibrate intermediate feature maps before dense feature processing. The second CBAM operates on the BiLSTM output to recalibrate sequence features before regression. These stages apply the same attention mechanism at different representation levels. Component ablations examine their individual and combined contributions. In addition, t-SNE and SHAP are used to visualize feature representations and assess feature contributions to model predictions. The main contributions of this paper are as follows:

(1) A spectral-feature-guided AVMD parameter-selection strategy is developed. It combines signal-informed candidate generation with a coarse-to-fine search and reconstruction-based screening and ranking. Its computational cost, parameter variability, and effect on wear prediction are evaluated against alternative VMD optimization methods.
(2) A two-stage attention architecture is constructed within an MSC–DenseNet–BiLSTM framework. CBAM modules are positioned before DenseNet and after BiLSTM to recalibrate intermediate and sequence representations, respectively. Controlled component ablations assess the contribution of each attention stage and the other principal network components.
(3) The framework is evaluated using TC18 milling data and the public PHM 2010 dataset, with comparisons against conventional predictors, TCN, and Transformer models. Raw-signal and VMD-based input comparisons, component ablations, and computational-cost measurements provide complementary assessments. Within-tool and exploratory cross-tool results are reported separately, while t-SNE and SHAP provide additional analysis of the learned representations and model predictions.

The remainder of this paper is organized as follows. Section 2 describes the overall framework and methodology. Section 3 presents the experimental design. Section 4 provides the results and analysis. Section 5 concludes the paper.

## 2. Overall Framework

### 2.1. Overall Framework

**Figure 1:**
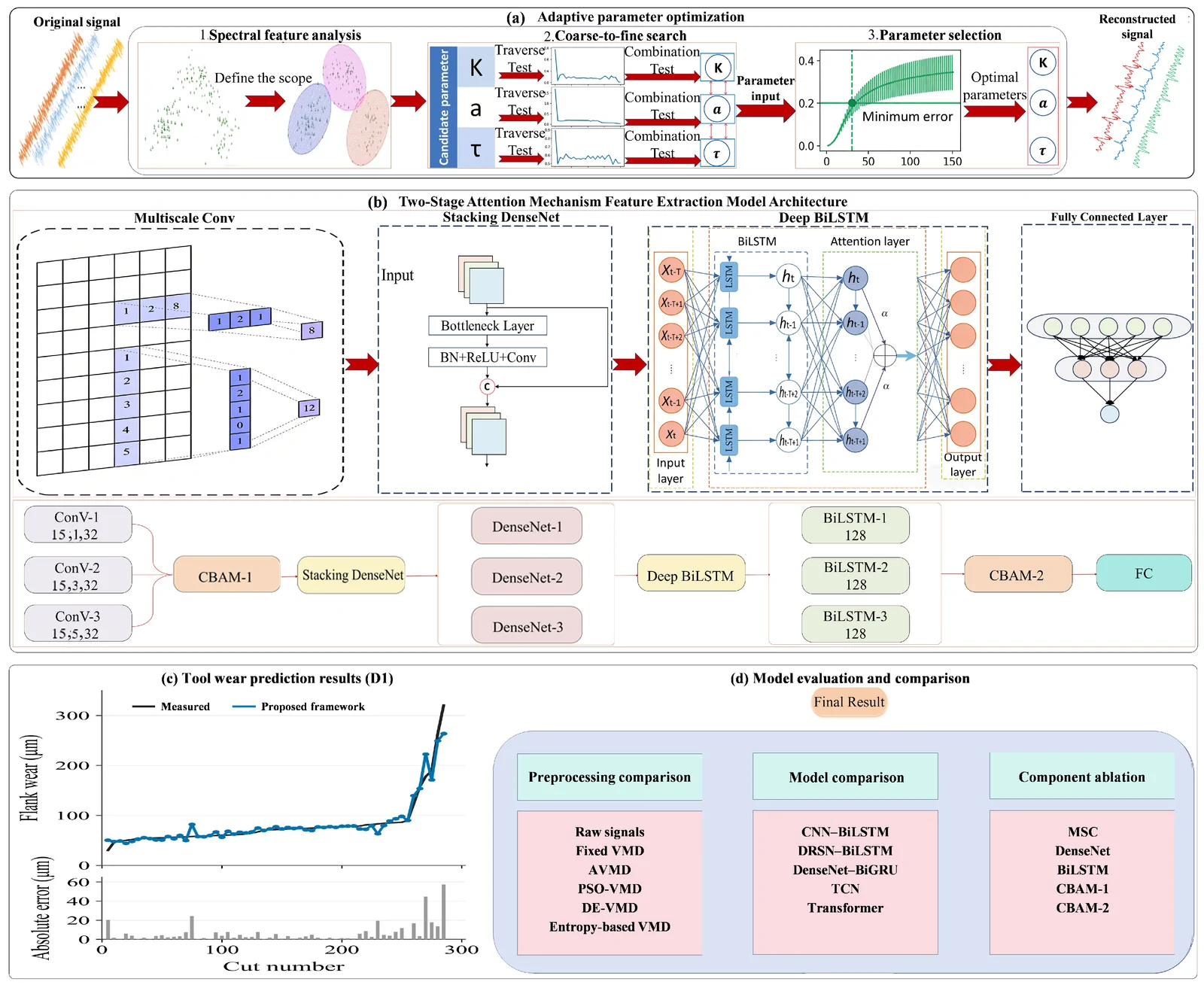
Overall framework for tool wear prediction: (**a**) spectral-feature-guided AVMD parameter selection; (**b**) two-stage attention-based feature extraction architecture; (**c**) tool wear prediction results; (**d**) model evaluation and comparison.

The proposed framework integrates Adaptive Variational Mode Decomposition (AVMD) with multiscale convolution, DenseNet, BiLSTM, and two-stage attention for multi-sensor tool wear prediction. The acquired sensor signals undergo stable-segment extraction, sequence-length standardization, and downsampling. AVMD decomposes and reconstructs the selected waveform channels, and the reconstructed signals are passed to the prediction network. In the reference two-stage architecture, the first CBAM follows multiscale convolution and precedes DenseNet, while the second follows BiLSTM. The resulting features are used to estimate tool flank wear, expressed in micrometres. Figure 1 illustrates the overall framework, which comprises three principal modules.

(1) Signal Preprocessing: Machining signals and their corresponding wear labels are organized according to the dataset and experimental condition. Waveform preprocessing includes stable-segment extraction, sequence-length standardization, and downsampling according to the recorded acquisition and processing settings. AVMD uses signal characteristics to guide parameter selection and decomposes the selected waveform channels into IMF components. Components are selected for reconstruction according to their absolute Pearson correlation with the input signal, following the selection criterion and fallback rule specified in Section 2.2.1. The reconstructed signals are then passed to the subsequent feature extraction module.
(2) Two-stage attention-based feature extraction: The reconstructed signals first pass through multiscale convolutional branches with different effective receptive fields. The first CBAM recalibrates the resulting feature maps through channel and spatial attention before they enter DenseNet. Dense connections then support feature propagation and reuse. The extracted features are processed by BiLSTM to capture bidirectional dependencies within the input signal sequence. The second CBAM recalibrates the BiLSTM output before regression. For implementations containing an additional engineered-feature branch, the engineered features are concatenated with the learned representation before entering the regression head. A wear estimate is produced after the complete input signal window for the corresponding cutting pass has been acquired and preprocessed.
(3) Prediction and model analysis: The regression head maps the extracted features to the corresponding tool wear value. The framework is evaluated using TC18 milling data and the public PHM 2010 dataset, with within-tool and cross-tool evaluation procedures described separately. Prediction performance is quantified using RMSE, MAE, and R^2^. Model comparisons, input comparisons, component ablations, and computational-cost measurements are used to assess different aspects of the framework. In addition, t-SNE visualizes feature representations, while SHAP assesses the contributions of the explained features to model predictions.

### 2.2. Methodology

#### 2.2.1. Adaptive Parameter Method for Variational Mode Decomposition (AVMD)

Variational Mode Decomposition (VMD) is an adaptive signal decomposition method based on a variational optimization framework. It aims to decompose a non-stationary signal into a set of intrinsic mode functions (IMFs) with narrowband characteristics. Each IMF is centered at a specific frequency, and the total bandwidth is minimized [43].

The core mathematical model of VMD is formulated as a constrained variational problem. The original signal *f*(*t*) is decomposed into *K* modal components {*u_k_* (*t*)}. The bandwidth of each component is estimated using the Hilbert transform and frequency shifting. The objective is to minimize the sum of the bandwidths of all modes, subject to the constraint $\sum_{k=1}^{k}u_{k}\left(t\right)=f\left(t\right)$. The constrained variational model is expressed as follows:

(1) $$\min_{\left\{u_{k}\},\{\omega_{k}\right\}}\sum_{k=1}^{K}{\left\|\partial_{t}\left\{\left[\left(\delta (t)+\frac{j}{\pi t}\right)*u_{k}(t)\right]e^{-j\omega_{k}t}\right\}\right\|}_{2}^{2},\;s.t.\;\sum_{k=1}^{K}u_{k}(t)=f(t)$$

where *ω_k_* denotes the center frequency of the *k*-th mode, *δ*(*t*) represents the Dirac delta function, and ∂*t* denotes the time derivative.

To address this problem, an augmented Lagrangian function is introduced to convert the constrained optimization problem into an unconstrained form.

(2) $$L(\{u_{k}\},\{\omega_{k}\},\lambda )=\frac{\alpha }{2}\sum_{k=1}^{K}{\left\|\partial_{t}\left\{\left[\left(\delta (t)+\frac{j}{\pi t}\right)*u_{k}(t)\right]e^{-j\omega_{k}t}\right\}\right\|}_{2}^{2}+{\left\|f(t)-\sum_{k=1}^{K}u_{k}(t)\right\|}_{2}^{2}+\langle \lambda (t),\sum_{k=1}^{K}u_{k}(t)-f(t)\rangle$$

The modal spectra ${\hat{u}}_{k}(\omega )$, center frequencies *ω_k_* and Lagrangian multipliers *λ*(*t*) are updated iteratively using the Alternating Direction Method of Multipliers (ADMM). The update equation for the modal spectra is given as follows:

(3) $${\hat{u}}_{k}^{n+1}(\omega )=\frac{\hat{f}(\omega )-\sum_{i=1}^{k-1}{\hat{u}}_{i}^{n+1}(\omega )-\sum_{i=k+1}^{K}{\hat{u}}_{i}^{n}(\omega )-\frac{{\hat{\lambda }}^{n}(\omega )}{2}}{1+\alpha {\left(\omega -\omega_{k}^{n}\right)}^{2}},\;\omega \geq 0$$

The center frequency is updated based on the center of mass of the energy in the frequency domain:

(4) $$\omega_{k}^{n+1}=\frac{\int_{0}^{\infty }\omega {\left|{\hat{u}}_{k}^{n+1}(\omega )\right|}^{2}d\omega }{\int_{0}^{\infty }{\left|{\hat{u}}_{k}^{n+1}(\omega )\right|}^{2}d\omega }$$

The iteration continues until the convergence criteria are met:

(5) $$\left|\epsilon_{\mathrm{mach}}+\frac{1}{M}\sum_{k=1}^{K}\sum_{m=0}^{M-1}{\left|{\hat{u}}_{k}^{n+1}[m]-{\hat{u}}_{k}^{n}[m]\right|}^{2}\right|\leq \varepsilon$$

where *M* is the number of frequency bins after mirror extension, $\epsilon_{mach}$ denotes machine precision, and ε is the convergence tolerance. The modal spectra used in the iterations are represented on the one-sided spectrum with negative-frequency entries set to zero. Iterations also terminate when the prescribed iteration limit is reached. The VMD decomposition process involves several key parameters, including the number of modes K, the penalty factor α, and the dual-ascent step size τ. The performance of the method depends strongly on these parameters. The number of modes K determines the decomposition resolution. A low value leads to under-decomposition and mode aliasing, while a high value introduces redundant components. The penalty factor α controls the bandwidth of each IMF. The parameter *τ* controls the step size of the Lagrange-multiplier update and influences the enforcement of the reconstruction constraint and the numerical behavior of the iterations. Existing VMD parameter optimization methods mainly rely on heuristic algorithms, such as differential evolution and particle swarm optimization. These methods perform random searches over a wide parameter space without explicit guidance from signal characteristics. Although they enable automatic parameter selection, they do not fully utilize the statistical properties and spectral structure of the signal. As a result, a large number of parameter combinations must be evaluated, which increases computational cost and limits their applicability in online adaptive scenarios under varying operating conditions. To address these limitations, an adaptive VMD method with feature-guided parameter optimization is proposed. The method establishes a relationship between signal spectral features and decomposition parameters to guide the optimization process. The range of the number of modes K is determined based on the distribution of spectral peaks. The search range of the penalty factor α is defined according to the energy concentration in the main frequency bands. The bounds of the dual-ascent step size τ are specified based on signal-to-noise ratio characteristics. This strategy reduces the parameter search space and decreases the number of parameter evaluations while maintaining decomposition quality. Consequently, the proposed method provides signal-informed parameter calibration for the evaluated sensor channels and cutting conditions.

Reconstruction quality is evaluated in the normalized signal domain. Let $x_{i}$ denote the input waveform after mean removal and maximum-absolute-value normalization, and let $r_{i}$ denote the reconstruction obtained by summing the retained IMFs. The centered reconstruction is defined as $r_{i}^{c}=r_{i}-\bar{r}$, where $\bar{r}=N^{-1}\sum_{i=1}^{N}r_{i}$ and *N* is the signal length. The reconstruction RMSE follows the definition in Section 4.3, with the compared quantities replaced by $x_{i}$ and $r_{i}^{c}$. The threshold RMSE ≤ 0.1 applies to normalized waveform reconstruction. NMSE and SNR are calculated as follows:

(6) $$\mathrm{NMSE}=\frac{\sum_{i=1}^{N}{\left(x_{i}-r_{i}\right)}^{2}}{\sum_{i=1}^{N}x_{i}^{2}+\eta }$$

(7) $$\mathrm{SNR}=10\log_{10}\left(\frac{\sum_{i=1}^{N}x_{i}^{2}+\eta }{\sum_{i=1}^{N}{\left(x_{i}-r_{i}^{c}\right)}^{2}+\eta }\right)\;\mathrm{dB}$$

where $\eta =10^{-10}$ is a small constant used for numerical stability. A separate smoothing-based SNR estimate is used to generate the candidate values of *τ*. It is calculated using the same SNR expression, with the Savitzky–Golay-smoothed input replacing the reconstructed waveform. Since a noise-free reference is unavailable, these reconstruction metrics quantify agreement with the observed input signal.

The implementation procedure of the proposed AVMD method is described as follows:

Step 1: FFT-based spectral analysis is performed on the preprocessed signal. Let *A* denote the positive-frequency FFT amplitudes and *x*_0_ the mean-centered signal. The robust noise scale is estimated as *σ*_r_ = 1.4826 × median(|*x*_0_ − median(*x*_0_)|). The peak-height threshold is *Q*_60_(*A*) + 0.5*σ*_r_, clipped between *Q_20_*(*A*) and 0.9max(*A*), where *Q_p_* denotes the *p*th percentile. Detected peak frequencies are merged by single-linkage clustering with a distance threshold of twice the FFT frequency resolution, retaining the highest-amplitude peak in each cluster. If no peak is detected, the eight highest-amplitude frequency bins are used as fallback candidates. The mode-number candidates are restricted to integers between 2 and 8. The lower bound is obtained by rounding half the valid-peak count upward, and the upper bound by rounding 1.8 times the peak count downward. When the bounds differ by less than two, the upper bound is extended to two above the lower bound, subject to the maximum of 8.

The spectral bandwidth is determined from the frequency span over which the amplitude exceeds 10% of its maximum, bounded by the FFT frequency resolution and one-third of the frequency-scale setting. The baseline penalty factor is calculated by dividing the squared frequency-scale setting by this bandwidth and clipping the result to between 500 and 5000. The candidate interval extends from 30% to twice the baseline value, with its lower and upper bounds restricted to 500 and 5000, respectively. Hierarchical clustering reduces the initial candidates to at most six values.

The smoothing-based SNR estimate guides a decreasing sigmoid mapping centered at 10 dB with a scale of 5 dB to obtain a reference value for *τ*. Initial candidates extend 0.3 below and above this reference value and are restricted to between 0 and 0.95. SNR-dependent filtering and merging of nearby candidates reduce the set to at most five values.

In the TC18 reference AVMD implementation, candidate generation uses a configured frequency scale of 400 Hz and an FFT length of 1024. This configured scale is distinct from the nominal sampling rate recorded during preprocessing. And SNR smoothing uses a second-order Savitzky–Golay filter with a nine-sample window. Ten equally spaced integer *α* candidates are generated and deduplicated. When more than six remain after screening, Ward clustering with a threshold of 0.2*α*_0_ represents each cluster by its integer median, retaining at most six distinct values. If fewer than three candidates remain, screening is repeated over 0.2–2.5 times the initial candidate median. Eight initial *τ* candidates are rounded to three decimal places and filtered to *τ* ≤ 0.4 for SNR > 15 dB, *τ* ≥ 0.5 for SNR < 5 dB, and 0.1 ≤ *τ* ≤ 0.6 otherwise. Ascending candidates are retained only when separated from the last retained value by more than 0.03. Empty reduced sets use *α* = {500, 1000, 1500, 2000, 2500, 3000} and *τ* = {0.1, 0.2, 0.3, 0.4, 0.5}. For mean-centered signal energy below 10^−10^, the fallback grids use *K* = 2–8, ten equally spaced integer *α* values from 500 to 5000, and eight equally spaced *τ* values from 0 to 0.95.

Step 2: Before parameter evaluation, the already preprocessed TC18 waveforms are checked for the required length of 5000 samples. Truncation or edge-padding is applied only when necessary, followed by mean removal and maximum-absolute-value normalization. For two mean-centered signals, similarity is defined as *S* = 0.5|*ρ*| + 0.5[1 − |*E*_1_ − *E*_2_|/max(*E*_1_, *E*_2_)], where *ρ* is the Pearson correlation coefficient and *E*_1_ and *E*_2_ are their squared-signal energies with 10^−10^ added for numerical stability. The rows of the pairwise dissimilarity matrix, with entries 1 − *S*, are used as input profiles for Ward clustering. Clusters are obtained using a linkage-distance threshold of 0.15. Within each cluster, samples are ordered by identifier, and the sample at index floor(*m*/2), using zero-based indexing, is selected as the representative, where *m* is the cluster size.

Candidate parameter combinations are first evaluated in parallel using a relaxed VMD convergence tolerance of 0.01. Combinations satisfying SNR ≥ 8 dB and RMSE ≤ 0.1 are retained and ranked by decreasing SNR, followed by increasing RMSE and NMSE. If no combination satisfies both thresholds, the five highest-ranked combinations are retained as fallback candidates. The leading 70% of the ranked candidates, rounded down, are selected for precise evaluation, with at least two retained when available. The precise VMD convergence tolerance is set to 10^−7^.

Early termination is considered after at least 15 successful precise evaluations. The search stops when the stability counter reaches six or the low-NMSE counter reaches four. An evaluation is considered stable when the absolute NMSE change does not exceed 0.0015 or the relative change is below 0.15%. Results with NMSE no greater than 0.0015 increment both counters. An unstable result resets the stability counter, and both counters are reset whenever the best candidate is updated.

Step 3: The parameter records obtained from the representative samples are collected separately for each sensor channel within the corresponding data group. Parameter combinations satisfying SNR ≥ 8 dB and RMSE ≤ 0.1 are ranked by decreasing SNR, followed by increasing RMSE and NMSE. If no combination satisfies both thresholds, all available valid records are ranked using the same criteria. The highest-ranked combination is selected as the channel-specific parameter set.

Each signal is then decomposed using the selected parameters. IMFs are retained when the absolute Pearson correlation coefficient between the mode and the input signal is at least 0.1. If fewer than two modes satisfy this criterion, the two modes with the highest absolute correlations are retained. The retained IMFs are summed to obtain the reconstructed signal for subsequent feature extraction and tool wear prediction.

#### 2.2.2. Feature Extraction with Two-Stage Attention

Effective extraction of wear-sensitive features from multi-sensor signals remains challenging under high-noise and varying operating conditions. To address this issue, a two-stage feature extraction method based on a dual convolutional attention mechanism is proposed, with the main processing sequence of MSC–CBAM–DenseNet–BiLSTM–CBAM. Multi-sensor signals decomposed and reconstructed by AVMD are first input into a multiscale convolutional module to extract local features at different receptive fields. In the first stage, CBAM recalibrates the extracted feature maps through channel and spatial attention before they enter DenseNet, where dense connections support feature propagation and reuse. In the second stage, BiLSTM captures bidirectional dependencies within the input sequence, and a second CBAM refines its output through channel and spatial attention. The two attention modules enhance relevant feature channels and informative sequence positions at different processing stages, providing discriminative representations for subsequent tool wear prediction.

##### CBAM

**Figure 2:**
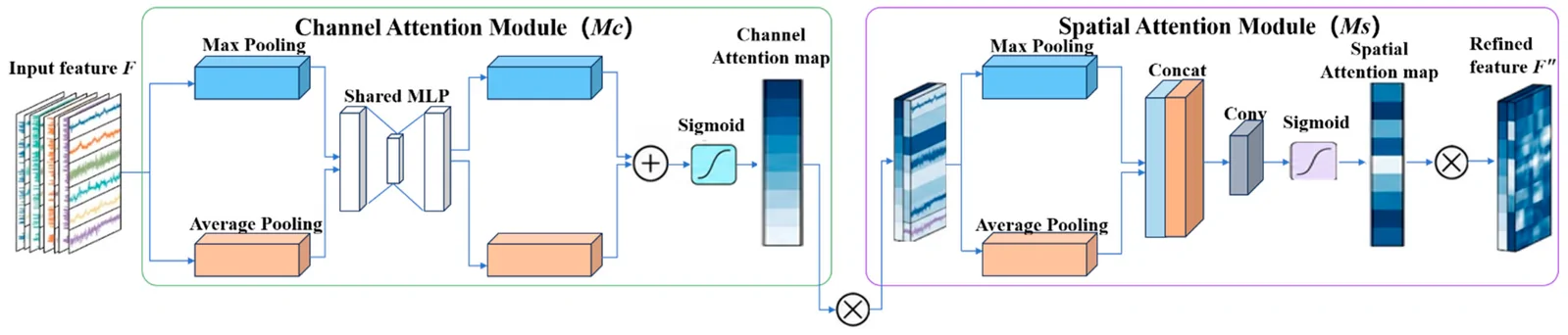
Channel attention and spatial attention structures in CBAM.

CBAM is a lightweight and general-purpose attention mechanism that achieves adaptive feature refinement by sequentially inferring attention weights along the channel and spatial dimensions [44]. Its structure is illustrated in Figure 2. CBAM first employs a channel attention submodule, which aggregates spatial information using global average pooling and max pooling. The aggregated features are then passed through a shared multi-layer perceptron to generate channel-wise weights, which are used to recalibrate the importance of different feature channels. Subsequently, the spatial attention submodule applies average pooling and max pooling across the channel dimension and concatenates the resulting descriptors. A one-dimensional convolution followed by batch normalization and sigmoid activation generates attention weights over sequence positions, which are multiplied with the channel-refined features. Through this sequential channel–spatial attention process, CBAM performs feature recalibration in two stages, which improves the model’s ability to capture and emphasize relevant information.

##### Multiscale Convolution (MSC)

**Figure 3:**
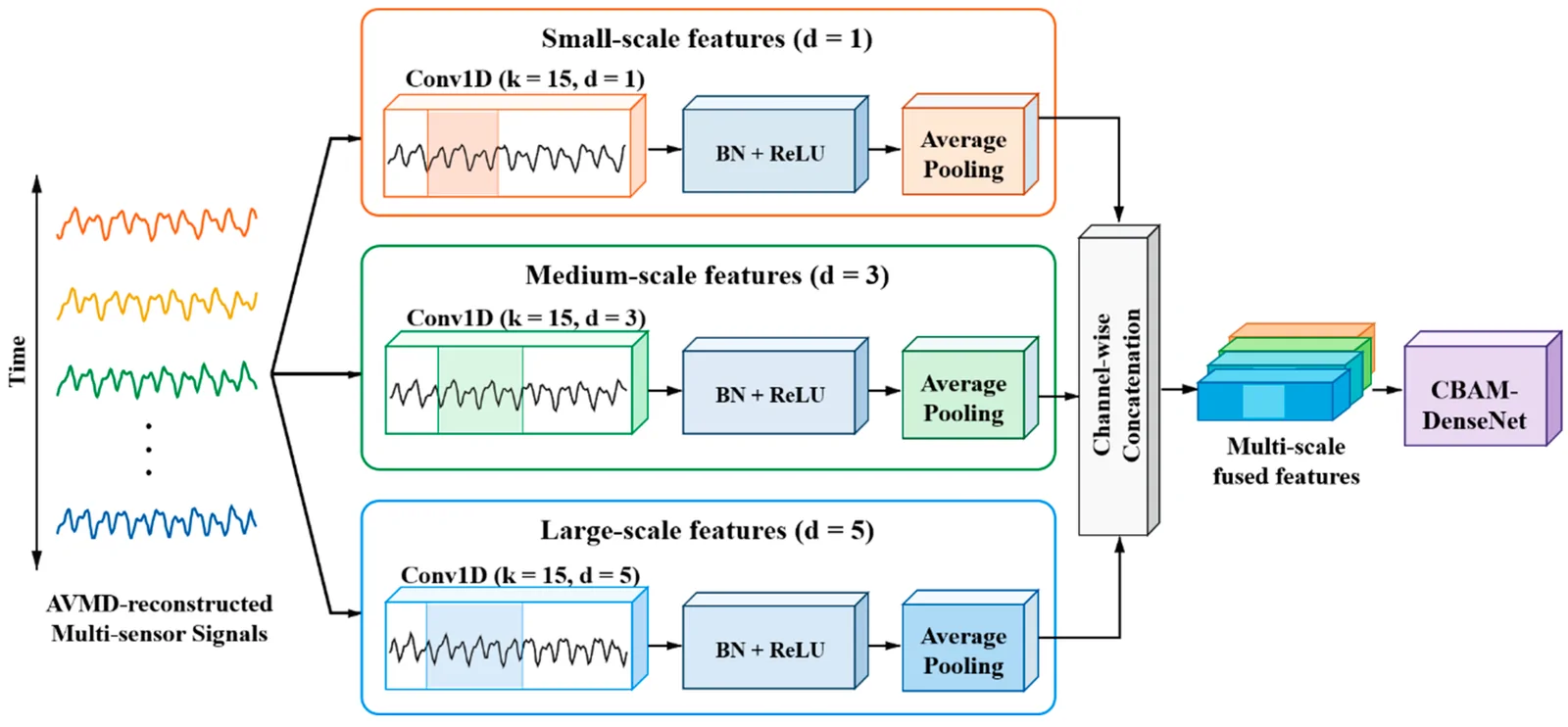
Multi-scale convolutional architecture.

To capture local signal patterns at different temporal scales, a multiscale convolutional module with three parallel branches is employed, as illustrated in Figure 3. Each branch uses a one-dimensional convolution with a kernel size of 15, while dilation rates of 1, 3, and 5 provide effective receptive fields of 15, 43, and 71 sampling points, respectively. Each convolution is followed by batch normalization and ReLU activation. Average pooling reduces the temporal length of the feature sequences and the computational cost of subsequent processing. The outputs of the three branches are concatenated along the channel dimension to form a fused multiscale representation for subsequent attention-based feature extraction.

##### DenseNet

**Figure 4:**
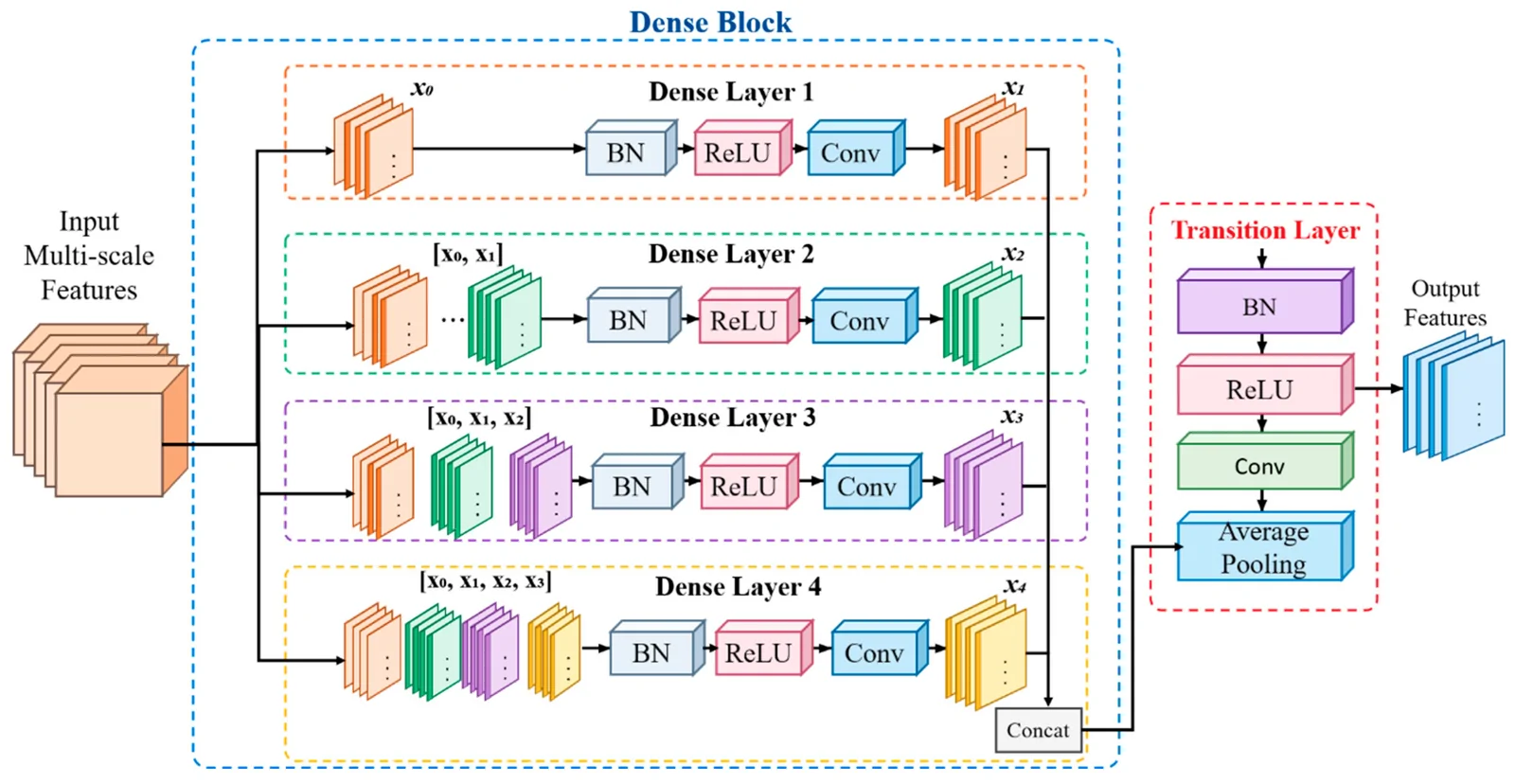
Schematic illustration of dense connectivity and a transition layer.

A one-dimensional DenseNet-based module is employed to further extract local features from the multiscale representations. Feature concatenation supports the reuse of information extracted at preceding processing stages. Figure 4 schematically illustrates dense connectivity and a transition layer. Its core advantage lies in the dense connection mechanism, as described in Equation (8). This mechanism enables each layer to receive feature maps from all preceding layers, which facilitates gradient propagation, promotes feature reuse, and improves parameter efficiency [45].

(8) $$x_{l}=H_{l}([x_{0},x_{1},\ldots ,x_{l-1}])$$

where $x_{0}$ denotes the input feature map, $x_{1},\ldots ,x_{l-1}$ denote the outputs of the preceding layers, and $\left[\cdot \right]$ denotes concatenation along the channel dimension. $H_{l}\left(\cdot \right)$ represents a composite function consisting of batch normalization (BN), rectified linear unit (ReLU), and one-dimensional convolution (Conv) operations. This dense connectivity structure mitigates the vanishing gradient problem through multiple information flow paths and promotes feature reuse across layers. The implemented module contains three densely connected blocks, with transition layers between adjacent blocks. Each block applies a BN–ReLU–Conv1D sequence with a kernel size of 1, followed by another BN–ReLU–Conv1D sequence with a kernel size of 3, and concatenates the resulting features with its input along the channel dimension. Transition layers use BN, ReLU, a pointwise convolution, and average pooling to compress the representations.

##### BiLSTM

**Figure 5:**
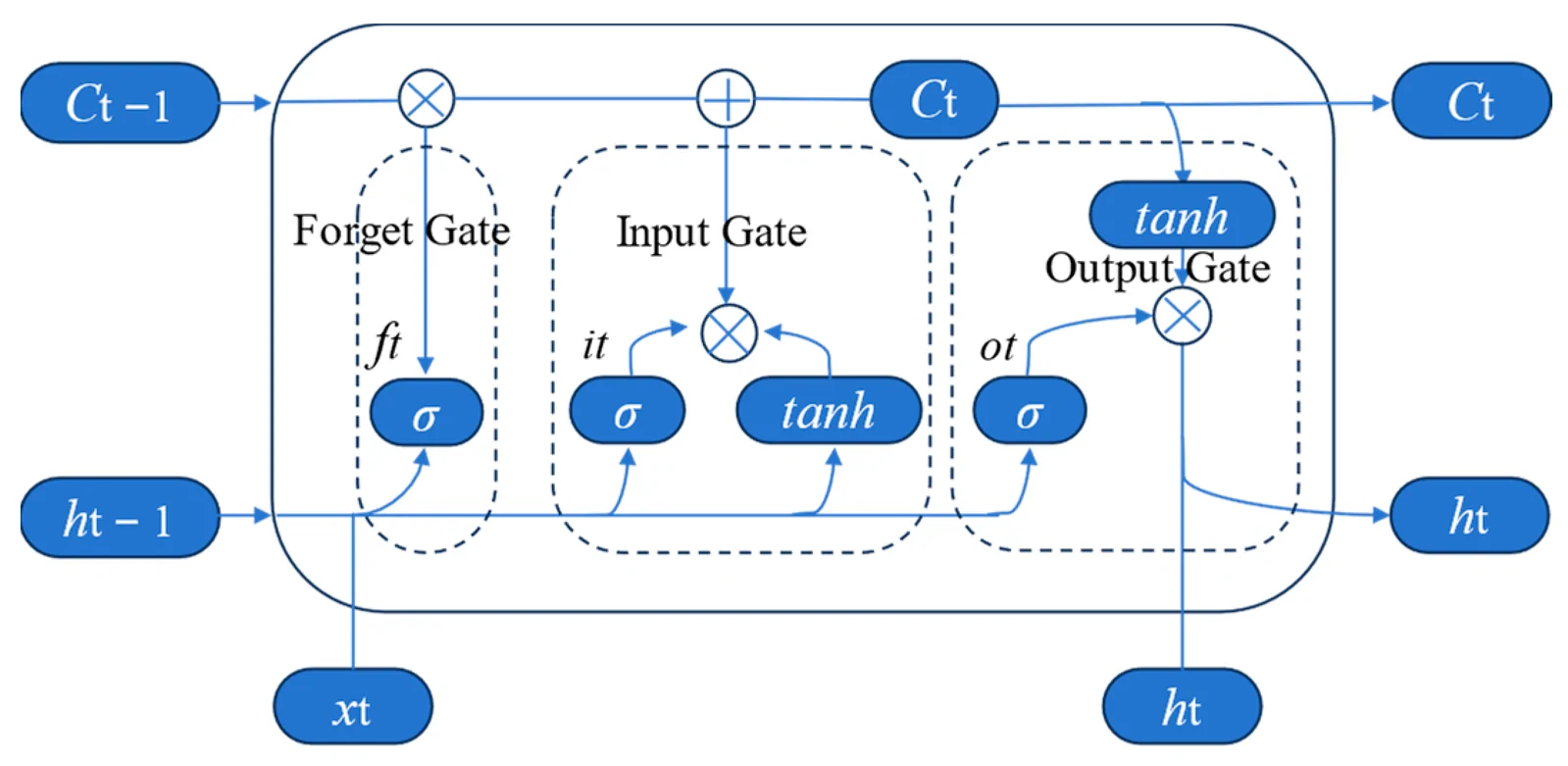
LSTM model framework.

LSTM mitigates the long-term dependency problem in recurrent neural networks through its gating mechanism. The internal state update process is illustrated in Figure 5.

In Figure 5, $x_{t}$ denotes the input at the current time step, while $c_{t}$ and $h_{t}\;$ represent the cell state and hidden state, respectively. The gate activations and state updates are calculated as follows:

(9) $$i_{t}=\sigma (W_{i}\cdot [h_{t-1},\;x_{t}]+b_{i})$$

(10) $$f_{t}=\sigma \left(W_{f}\cdot \left[h_{t-1},x_{t}\right]+b_{f}\right)$$

(11) $$o_{t}=\sigma \left(W_{o}\cdot \left[h_{t-1},x_{t}\right]+b_{o}\right)$$

(12) $${\tilde{c}}_{t}=tanh\left(W_{c}\cdot \left[h_{t-1},x_{t}\right]+b_{c}\right)$$

(13) $$c_{t}=f_{t}⊙c_{t-1}+i_{t}⊙{\tilde{c}}_{t}$$

(14) $$h_{t}=o_{t}⊙tanh\left(c_{t}\right)$$

where $i_{t}$, $f_{t}$, and $o_{t}$ denote the input, forget, and output gates, respectively; ${\tilde{c}}_{t}$ denotes the candidate cell state; and $c_{t}$ and $h_{t}$ denote the cell state and hidden state. W and b represent learnable weight matrices and bias vectors, respectively. The symbols σ, tanh, and ⊙ denote the sigmoid function, hyperbolic tangent function, and element-wise multiplication, respectively, while $\left[h_{t-1},x_{t}\right]$ denotes concatenation.

**Figure 6:**
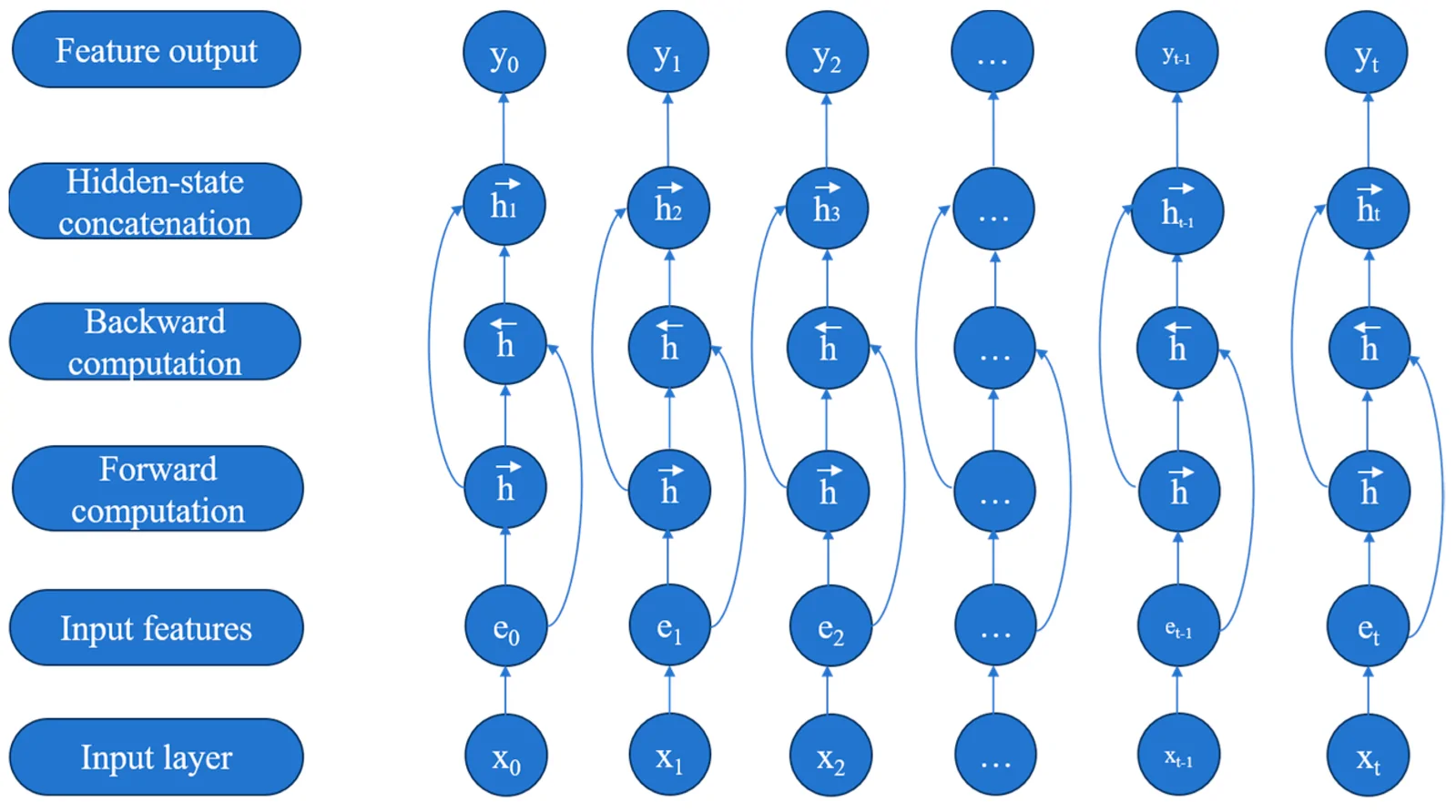
Principles of the BiLSTM algorithm.

A bidirectional long short-term memory network (BiLSTM) is employed to capture temporal dependencies in the feature sequence produced by the preceding modules. The forward and backward LSTMs process this sequence in opposite directions, and their hidden states are concatenated at each sequence position to form a bidirectional contextual representation. The implemented module contains three stacked BiLSTM layers, each with 128 hidden units per direction. Its output is subsequently refined by the second CBAM before downstream regression. Figure 6 illustrates the principle of bidirectional sequence processing.

##### Fully Connected Layer

**Figure 7:**
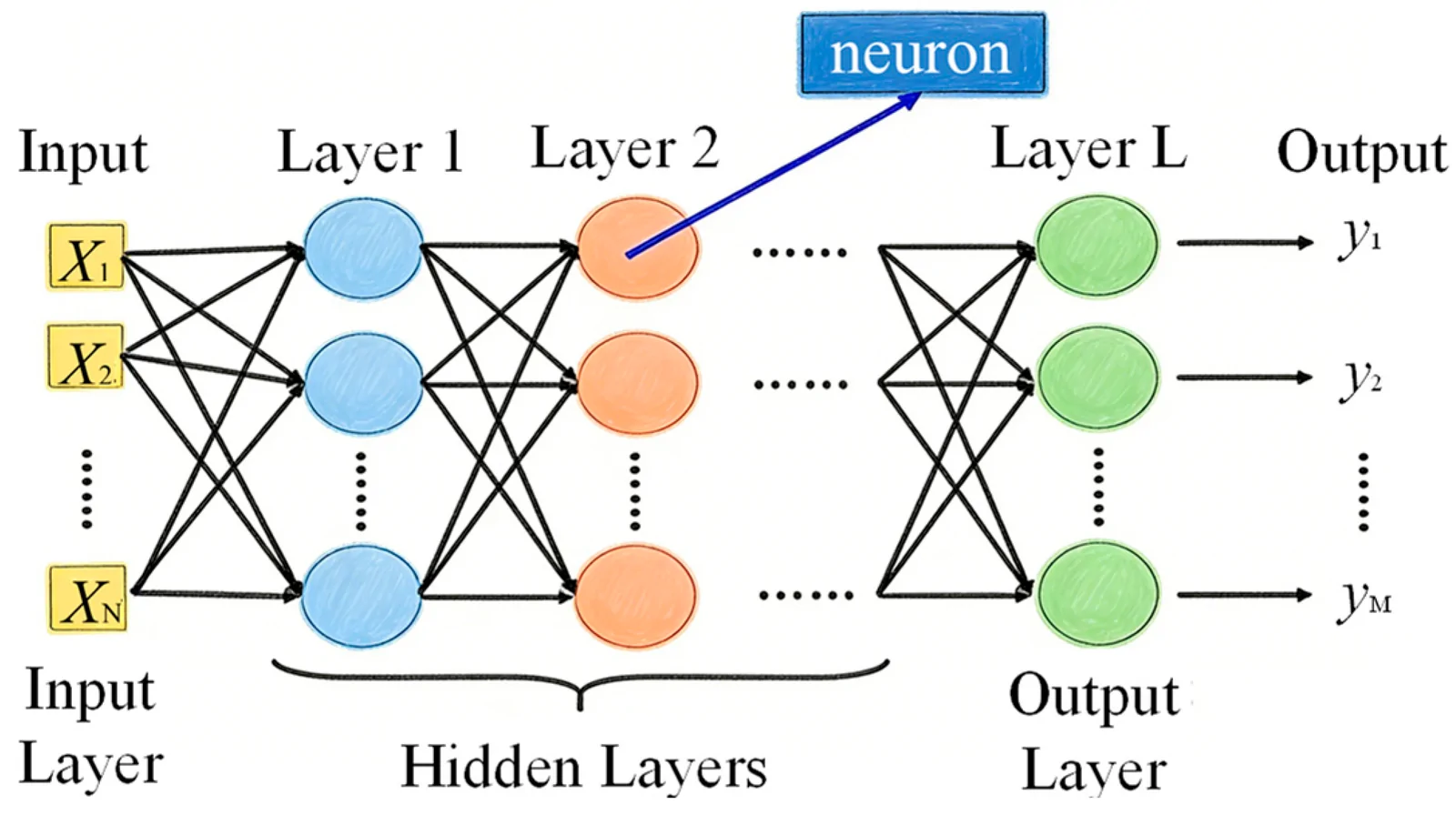
Schematic diagram of a fully connected layer.

The extracted feature representation is passed to a fully connected regression head to estimate tool flank wear. The regression head contains two hidden layers with 64 and 32 neurons, respectively, followed by a single output neuron. ReLU activation introduces nonlinearity between the hidden layers, while dropout is used during training to reduce overfitting. The final linear layer produces a continuous wear estimate expressed in micrometres. Figure 7 provides a schematic illustration of fully connected connections.

#### 2.2.3. Implementation of Two-Stage Attention-Based Feature Extraction

Using the multi-channel signal tensor reconstructed via AVMD ($X\in R^{C\times T}$, where C denotes the number of channels and T denotes the number of time steps) as input, feature extraction is performed using a two-stage attention mechanism. The specific procedure is as follows:

Step 1: The multi-sensor signals reconstructed by AVMD are input into a multiscale convolutional module containing three parallel branches. Each branch uses a one-dimensional convolution with a kernel size of 15, with dilation rates of 1, 3, and 5, respectively. Batch normalization, ReLU activation, and average pooling are applied in each branch. The branch outputs are aligned in sequence length and concatenated along the channel dimension to form the multiscale feature representation X, which is subsequently processed by the first CBAM.

Step 2: The first CBAM is placed after multiscale convolution and before DenseNet. Its channel attention submodule applies global average pooling and max pooling along the temporal dimension, followed by a shared transformation and sigmoid activation, to generate the channel attention weights $M_{c}$, as defined in Equation (15). The channel-refined representation is obtained as $X_{c}=X⊙M_{c}$, where ⊙ denotes element-wise multiplication with broadcasting. This operation adaptively recalibrates the learned feature channels.

(15) $$M_{c}=\sigma \left(\mathrm{MLP}\left(\mathrm{GlobalAvgPool}\left(X\right)\right)+\mathrm{MLP}\left(\mathrm{GlobalMaxPool}\left(X\right)\right)\right)$$

where $M_{c}$ denotes the channel attention weights, σ is the sigmoid function, and MLP denotes the shared channel transformation. GlobalAvgPool and GlobalMaxPool aggregate each feature channel along the temporal dimension.

Step 3: The channel-refined representation $X_{c}$ is processed by the spatial attention submodule. Average pooling and max pooling are performed across the feature channels, and the resulting descriptors are concatenated. A one-dimensional convolution with a kernel size of 7, followed by batch normalization and sigmoid activation, generates the attention weights $M_{s}$ over sequence positions, as defined in Equation (16). The refined representation is obtained as $X_{\mathrm{att}}=X_{c}⊗M_{s}$.

(16) $$M_{s}=\sigma \left(\mathrm{BN}\left(\mathrm{Conv}1D_{k=7}\left(\left[{\mathrm{AvgPool}}_{c}(X_{c});{\mathrm{MaxPool}}_{c}(X_{c})\right]\right)\right)\right)$$

where the subscript c denotes pooling across feature channels, and $\left[\cdot ;\cdot \right]$ denotes channel-wise concatenation.

Step 4: The attention-refined features $X_{att}$ are passed to the stacked DenseNet module for further feature extraction and reuse. The module contains three blocks, each concatenating its transformed features with its input along the channel dimension. Transition layers between adjacent blocks apply batch normalization, ReLU activation, pointwise convolution, and average pooling to compress the representations. The resulting feature representation *F* is organized along the sequence and feature-channel dimensions for subsequent temporal modeling.

Step 5: The feature representation *F* is reshaped and passed to the stacked BiLSTM module to capture bidirectional dependencies within the input feature sequence. The second CBAM then refines the BiLSTM output through sequential channel and spatial attention, adaptively recalibrating feature channels and sequence positions. The refined features are passed to the subsequent pooling and regression stages to produce a continuous estimate of tool flank wear in micrometres.

**Figure 8:**
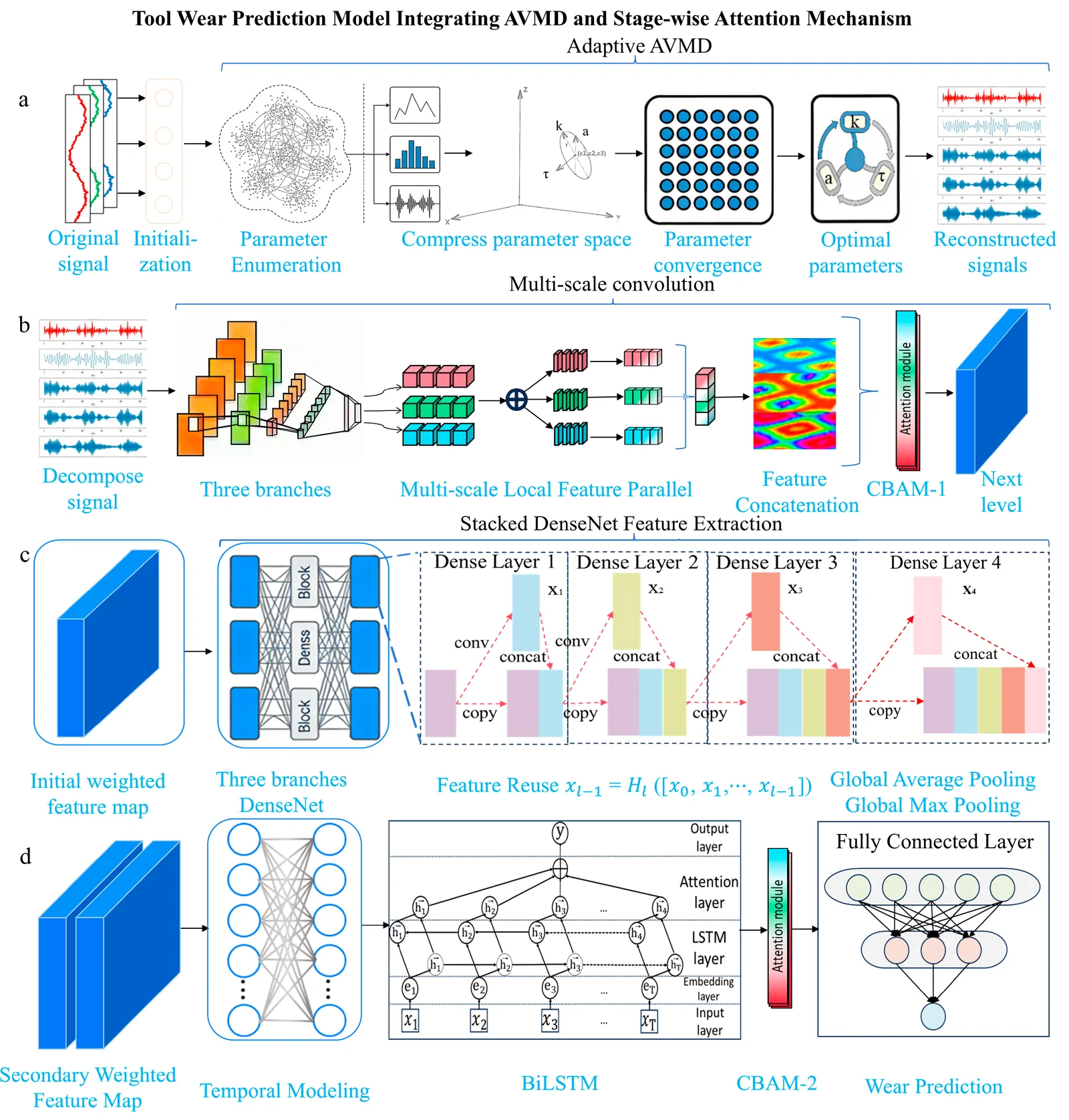
Tool wear prediction model integrating AVMD and two-stage attention: (**a**) adaptive signal preprocessing; (**b**) multiscale convolution and the first CBAM; (**c**) dense feature processing with feature reuse; (**d**) BiLSTM temporal modeling, the second CBAM, and wear regression. Engineered features are concatenated before regression when enabled.

The overall structure of the model is shown in Figure 8, and the complete algorithmic workflow is described in Algorithm 2.
**Algorithm 2:** Tool Wear Prediction Integrating Adaptive VMD and Two-Stage Attention**Input:** AVMD-reconstructed tensor *X* (batch × time × sensor channels); engineered features *g* when used; trained parameters. **Output:** Predicted tool flank wear *y*_pred_ (μm). 1   **Function** ToolWearPredict(*X*, *g*) 2       Set the trained network to evaluation mode; use stored BN statistics and disable dropout for prediction. 3       F ← Permute(*X*) to batch × channels × time. 4       for *d* in {1, 3, 5} do 5       *B*_d_
*←* Conv1D(*F*, output channels = 32, kernel = 15, dilation = *d*, stride = 1, padding = *p*_*d*), where p_1_ = 7, p_3_ = 14, and p_5_ = 21. 6       *B*_d_ ← ReLU(BN(*B*_d_)). 7       Align *B*_d_ to 2500 temporal positions by retaining the first 2500 positions when longer, or appending zeros when shorter. 8       *B*_d_ ← AvgPool1D(*B*_d_, kernel = 2, stride = 2). 9       end for 10     F ← Concatenate(*B*_1_, *B*_3_, *B*_5_) along channels, giving 96 feature channels. 11     F ← CBAM(*F*; first-stage parameters). 12     for block index *j* from 1 to 3 do 13       H ← Conv1D(ReLU(BN(*F*)), output channels = 128, kernel = 1). 14       H ← Conv1D(ReLU(BN(*H*)), output channels = 32, kernel = 3, padding = 1). 15       F ← Concatenate(*F*, *H*) along the channel dimension. 16       if *j* < 3 then 17         F ← Conv1D(ReLU(BN(*F*)), output channels = floor(channels(*F*)/2), kernel = 1). 18         F ← AvgPool1D(*F*, kernel = 2, stride = 2). 19       end if 20     end for 21     F ← ReLU(BN(*F*)). 22     Q ← Permute(*F*) to batch × time × channels. 23     If the temporal length of *Q* is odd, discard its final position; fold adjacent pairs into channels (factor = 2). 24     for recurrent layer *l* from 1 to 3 do 25       Q ← BiLSTM(*Q*, hidden units = 128 per direction); concatenate forward and backward hidden states. 26       Q ← Dropout(*Q*, rate = 0.3). 27     end for 28     F ← CBAM(Permute(*Q*) to batch × channels × time; second-stage parameters). 29     Q ← Unfold(Permute(*F*) to batch × time × channels, factor = 2). 30     v ← GlobalAveragePool(*Q*) along the temporal dimension. 31     v ← Dropout(*v*, rate = 0.3). 32     If the engineered-feature branch is enabled, *v* ← Concatenate(*v*, *g*) along the feature dimension. 33     h ← Dropout(ReLU(Linear(*v*, 64)), rate = 0.3). 34     h ← ReLU(Linear(*h*, 32)). 35     *y*_pred_ ← Linear(*h*, 1). 36     return *y*_pred_. 37   **End Function** 38   **Function** CBAM(*F*; stage-specific parameters) 39     *A*_avg_ ← GlobalAveragePool(*F*); *A*_max_ ← GlobalMaxPool(*F*), both along the temporal dimension. 40     *M*_c_ ← Sigmoid(MLP(*A*_avg_) + MLP(*A*_max_)) using the shared channel transformation [Equation (15)]. 41     *F*_c_ ← *F* ⊙ *M*_c_, with broadcasting over sequence positions. 42     *S*_avg_ ← Mean(*F*_c_); *S*_max_ ← Max(*F*_c_), both across feature channels at each sequence position. 43     *M*_s_ ← Sigmoid(BN(Conv1D(Concatenate(*S*_avg_, *S*_max_), output channels = 1, kernel = 7, padding = 3))). 44     return *F*_c_ ⊙ *M*_s_, with broadcasting across feature channels [Equation (16)]. 45   **End Function**

## 3. Experimental Setup and Datasets

This study evaluates the proposed framework using an in-house TC18 titanium-alloy milling dataset collected under three distinct cutting conditions and the publicly available PHM 2010 milling dataset collected under fixed cutting parameters. The two datasets are used to assess tool wear prediction performance under different experimental settings.

### 3.1. TC18 Milling Experiment

**Figure 9:**
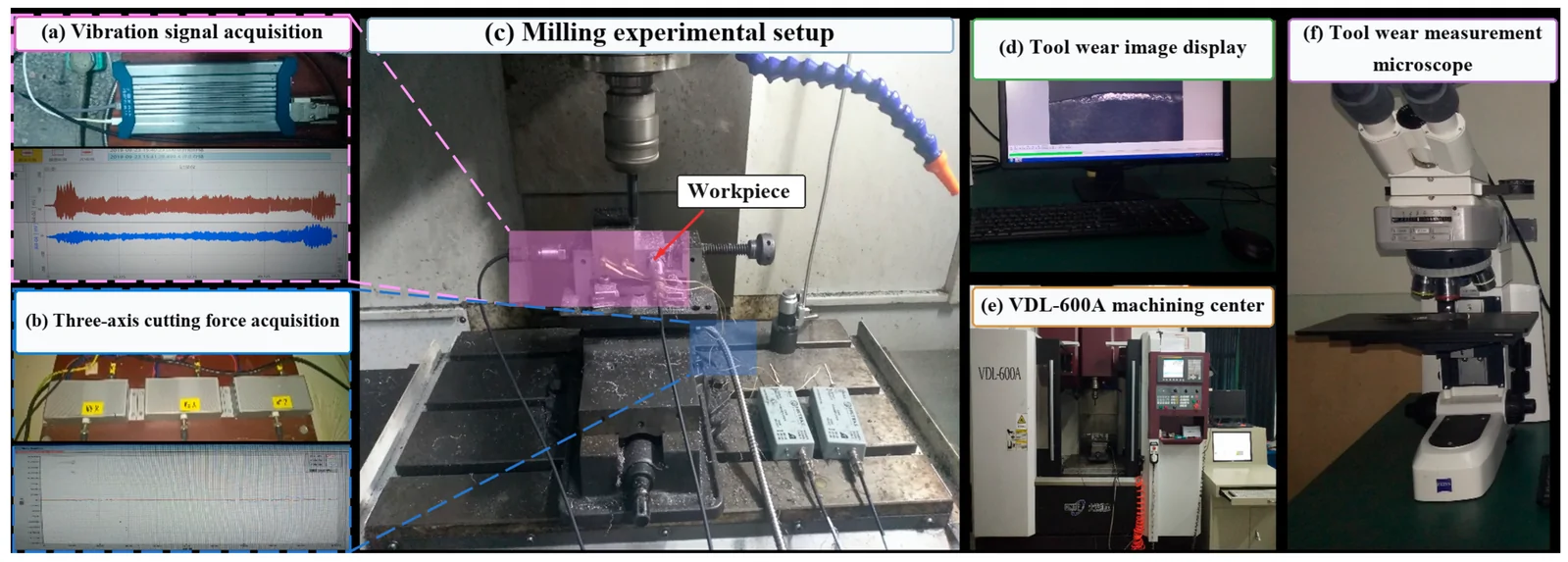
Experimental setup and measurement system: (**a**) vibration signal acquisition; (**b**) three-axis cutting force acquisition; (**c**) milling experimental setup; (**d**) tool wear image display; (**e**) VDL-600A machining center; and (**f**) tool wear measurement microscope.

**Figure 10:**
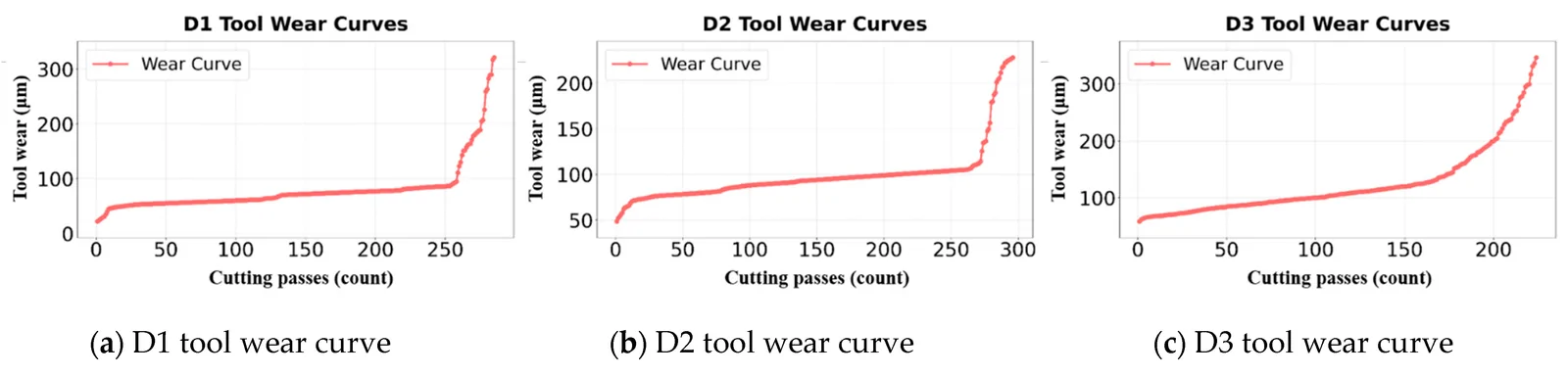
Measured tool wear curves under the three TC18 milling conditions: (**a**) D1; (**b**) D2; and (**c**) D3.

The TC18 milling experiment comprised three tool-life tests under three distinct cutting conditions, with one fresh tool tested under each condition. Machining was performed on a VDL-600A vertical machining center (Dalian Machine Tool Group, Dalian, China) using a TC18 titanium alloy workpiece and a 16 mm cutter body (TAP300D16-C16-150-2T) fitted with two APMT1135 carbide inserts. The machined surface dimensions of the workpiece were 60 mm × 80 mm, with the 60 mm dimension representing the feed direction. Cutting force signals (*F_x_*, *F_y_*, *F_z_*) were collected using a YDCB-III05B piezoelectric three-axis force measurement system, with the force gauge mounted on the bottom of the fixture vise on the machine tool table. Vibration signals in the *x* and *y* directions were acquired using a DH5901 measurement system (Shanghai Donghao Test Technology Co., Ltd., Shanghai, China) equipped with piezoelectric accelerometers mounted on the front and side of the fixture, respectively. Acoustic-emission data were acquired using a PCI-8 system (Physical Acoustics Corporation, Princeton Junction, NJ, USA), with the probe attached to the front surface of the workpiece. AEwin software recorded amplitude, energy, counts, duration, and rise time. Flank wear was measured offline after each cutting cycle, which comprised two passes along the 60 mm feed direction. A Zeiss AXIO SCOPE A1 microscope (Carl Zeiss Microscopy GmbH, Jena, Germany) with a 50× objective and ProImaging software were used for measurement. The flank-wear region was evaluated at half the axial cutting depth, and the maximum width of the flank-wear band was measured on each insert. The tool-level wear value, VB, was defined as the arithmetic mean of the measurements from the two inserts. The supplied wear-label sequence was used as the regression target, expressed in micrometres. Both the cutting force and vibration sensors had a sampling frequency of 20 kHz. The software version identifiers and the manufacturer of the YDCB-III05B system were not specified in the available archived experimental documentation. A schematic of the experimental setup is presented in Figure 9. Process parameters were selected according to the recommended ranges provided by the tool manufacturer, with specific values listed in Table 1. Tool wear curves measured during the TC18 machining process under the three operating conditions are displayed in Figure 10.

The D1, D2, and D3 datasets contained 285, 296, and 224 labeled records, respectively. Under the every-fifth-record evaluation split described in Section 4.2, the evaluation subsets contained 57, 59, and 44 records, leaving 228, 237, and 180 records, respectively, outside the evaluation subsets.

### 3.2. PHM 2010 Dataset

**Figure 11:**
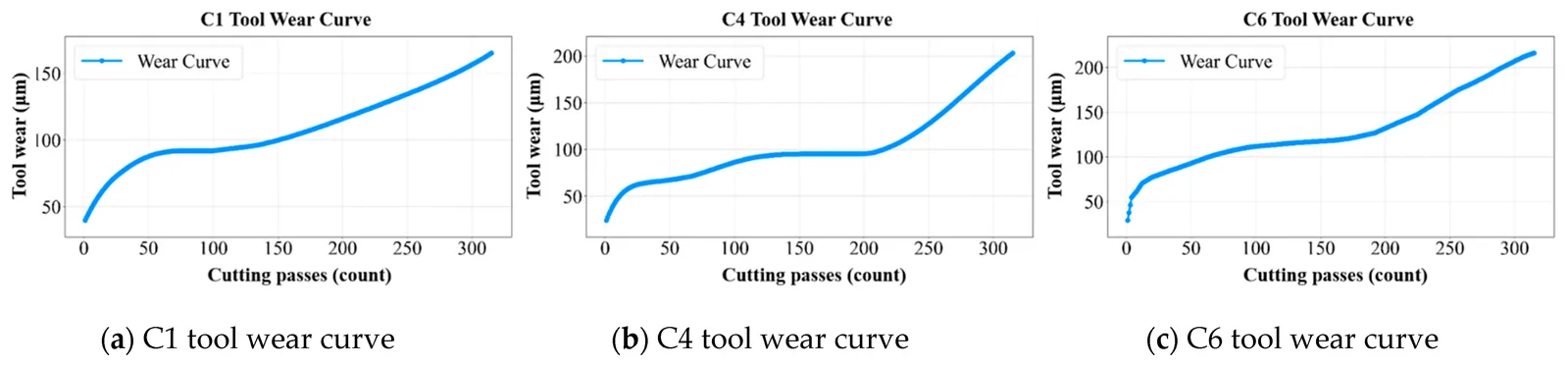
Mean flank wear curves for the PHM 2010 dataset: (**a**) C1; (**b**) C4; and (**c**) C6.

The PHM 2010 experimental data were obtained from the publicly available dataset of the 2010 PHM Society Conference Data Challenge [46]. This dataset utilized a *R*öders Tech RFM760 high-speed CNC machine tool (*R*öders GmbH, Soltau, Germany) as the machining platform to perform dry milling operations under consistent operating conditions throughout the service life of six cutting tools. The workpiece material was stainless steel with a hardness of HRC 52, and the cutting tools were three-fluted ball-nose carbide end mills. A Kistler 3D force transducer (Kistler Instrumente AG, Winterthur, Switzerland) was installed between the workpiece and the machining table to collect cutting force data. Concurrently, Kistler accelerometers and acoustic emission sensors (Kistler Instrumente AG, Winterthur, Switzerland) were mounted on the workpiece to acquire vibration and acoustic emission signals, respectively. The public data records contain seven channels: three cutting-force components, three vibration components, and one acoustic-emission root-mean-square (AE-RMS) channel. All seven channels were acquired at a sampling frequency of 50 kHz. The milling parameter settings are provided in Table 2. Each tool record contains 315 consecutive cuts, with flank wear measured offline using a LEICA MZ12 microscope (Leica Microsystems, Heerbrugg, Switzerland). Public wear labels are available for tools C1, C4, and C6; therefore, these three records were selected for evaluation. For each cut, the arithmetic mean of the flank wear values measured on the three cutting edges was used as the regression target, expressed in micrometres. The wear curves for these three tools are shown in Figure 11.

## 4. Results and Discussion

### 4.1. AVMD Signal Decomposition Results

In the TC18 preprocessing pipeline, stable cutting segments were extracted from the force and vibration signals acquired at 20 kHz. Candidate windows containing 10,000 samples were screened based on the lowest 20% of window variances, and the window with the smallest variation among its 2000-sample subwindow variances was selected. The selected signals were processed using a fourth-order Butterworth low-pass filter with forward–backward filtering and subsequently reduced to 5000 samples through equidistant sampling, corresponding to a nominal sampling rate of 10 kHz and a filter cutoff frequency of 4 kHz. Before AVMD processing, the signals were mean-centered and normalized by their maximum absolute amplitudes, with sequence-length correction applied when necessary. Channel-specific AVMD parameters were determined using representative signals and applied to the corresponding sensor channels. Table 3 summarizes the parameter combinations (*K*, *α*, *τ*) recorded in the archived AVMD files used for the TC18 prediction experiments.

Additional experiments compared raw signals, fixed-parameter VMD, AVMD, PSO-VMD, DE-VMD, and envelope-entropy VMD. All VMD parameter optimization methods used the same search bounds of *K* = 2–8, *α* = 500–5000, and *τ* = 0–0.95. AVMD employed signal-dependent candidate sets and followed the screening and ranking procedure described in Section 2.2.1 and Algorithm 1, using SNR ≥ 8 dB and RMSE ≤ 0.1 as reconstruction criteria. The convergence tolerances for coarse and precise evaluations were 0.01 and 10^−7^, respectively, with a maximum of 500 iterations per decomposition.

For the comparison optimizers, the population size was set to 6, with random seeds of 42, 43, and 44. PSO employed an inertia weight of 0.7 and cognitive and social coefficients of 1.5, while DE used a mutation factor of 0.7 and a crossover probability of 0.8. PSO-VMD and DE-VMD adopted the mean composite reconstruction score as their fitness function, whereas envelope-entropy VMD used envelope entropy as its optimization objective.

Table 4 summarizes the parameter calibration costs on the TC18 and PHM 2010 datasets. Calibration times were calculated by summing the search times of individual sensor channels. Each parameter-search method was evaluated through three complete searches, and the results are reported as the mean ± sample standard deviation.

Each TC18 search involved 822 VMD evaluations, while the PHM 2010 experiments used 2370 evaluations for AVMD and 2358 for the comparison methods. Under these closely matched evaluation budgets, AVMD achieved the shortest calibration time among the evaluated optimization methods, demonstrating favorable computational efficiency in parameter selection across the tested configurations.

Reconstruction agreement was evaluated separately for each sensor channel using RMSE, NMSE, and SNR. Metrics were calculated for individual test samples and then averaged within the same channel. Table 5 presents the retained-mode reconstruction results for *F_x_* as a representative channel across D1–D3. Reconstruction metrics in Table 5 were calculated after mean removal on the stored supplementary waveform scale, before network-input standardization. No additional maximum-absolute-value normalization was applied during this metric calculation. These RMSE values are distinct from wear-prediction errors expressed in micrometres.

As shown in Table 5, AVMD demonstrated competitive reconstruction performance across the three TC18 cutting conditions, with particularly favorable results under D2 and D3. For the representative *F_x_* channel, AVMD achieved the lowest RMSE values of 0.00371 and 0.01004 under D2 and D3, respectively, corresponding to reductions of 81.6% and 49.4% compared with fixed-parameter VMD. The corresponding SNR values reached 44.42 dB and 56.33 dB, with the latter matching the highest value among the compared methods. Although fixed-parameter VMD performed better under D1, the results indicate that AVMD can achieve high reconstruction agreement under different cutting conditions.

The repeatability of parameter selection was evaluated through three independent executions of each optimization method. Table 6 presents the selected parameters for the representative *F_x_* channels of TC18 D1 and PHM 2010 C6. The results are reported as the mean ± sample standard deviation of the three searches, providing a quantitative assessment of parameter-selection stability.

The randomized searches produced variations in the selected parameter combinations. For example, the mode counts for TC18 D1 *F_x_* were 5.00 ± 3.00, 3.00 ± 1.73, and 6.33 ± 1.15 for PSO-VMD, DE-VMD, and envelope-entropy VMD, respectively. In contrast, all three AVMD searches selected *K* = 4, *α* = 1867, and *τ* = 0.183 for this channel. Across the two calibration settings, AVMD returned identical parameter combinations in all three repetitions for each of the 12 channels.

**Figure 12:**
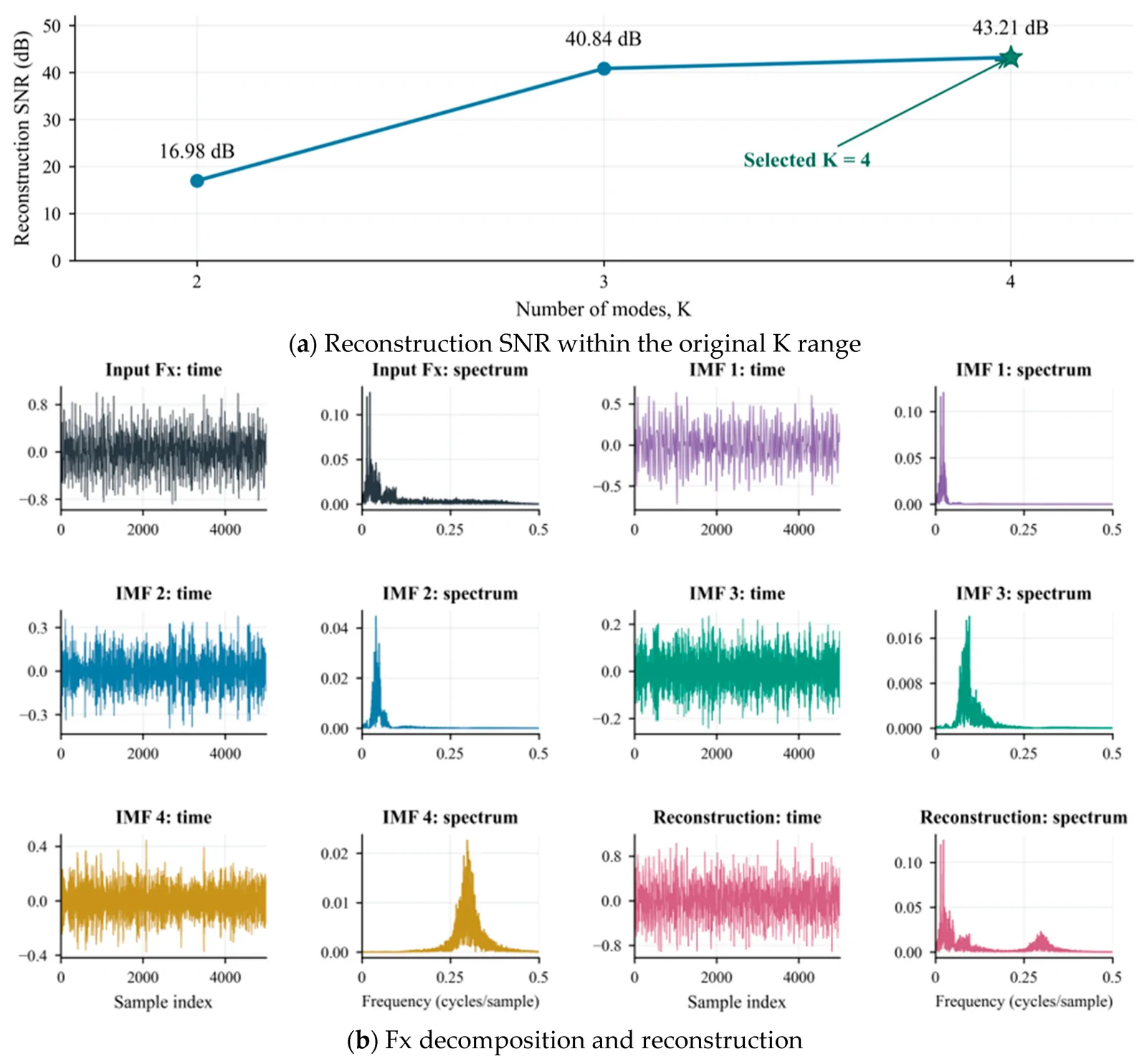
AVMD mode-count analysis and signal decomposition for TC18 D1 Fx: (**a**) reconstruction SNR for different mode counts; (**b**) time-domain waveforms and amplitude spectra of the input signal, IMFs, and reconstruction.

Figure 12a presents a controlled comparison of reconstruction SNR within the original candidate range of *K* = 2–8 for the D1 *F_x_* calibration signal at cut 1, with *α* = 1867 and *τ* = 0.183 held constant. *K* = 4 achieved the highest reconstruction SNR within this range, consistent with the parameter combination selected by the original screening and ranking procedure. Figure 12b presents the input *F_x_* signal, four retained IMFs, and their reconstruction for cut 107 under condition D1. The selected parameters were *K* = 4, *α* = 1867, and *τ* = 0.183, and all four IMFs were retained for signal.

To evaluate the effects of different signal preprocessing methods, all variants were compared using the same prediction architecture, engineered features, data partition, and training settings. Consistent experimental conditions were maintained across the compared methods to ensure a fair assessment of their contributions to tool wear prediction performance.

As shown in Table 7, AVMD achieved the best prediction performance under D2, with an RMSE of 7.4098 μm, an MAE of 4.7217 μm, and an R^2^ of 0.9470, substantially outperforming the other preprocessing methods. Under D1, AVMD achieved a competitive R^2^ of 0.9399, slightly below the highest value of 0.9416 obtained by DE-VMD. Under D3, raw signals yielded the highest R^2^ of 0.9665. These results indicate that the effectiveness of signal preprocessing varies across cutting conditions, and improved reconstruction agreement does not necessarily translate into higher downstream wear-prediction accuracy.

### 4.2. Feature Extraction Analysis Using Two-Stage Attention

This section examines feature extraction using the reference architecture containing multiscale convolution, DenseNet, BiLSTM, and two-stage CBAM attention, with condition D1 used as an example. The first CBAM refines the multiscale features before DenseNet, while the second recalibrates the BiLSTM output. The reference architecture and base-stage training settings are summarized in Table 8.

Samples were arranged according to their cutting-pass numbers and divided using a systematic 4:1 partitioning strategy. Specifically, within each group of five consecutive cutting passes, the first four samples were assigned to the training subset, while the fifth was used for evaluation, resulting in an approximately 80:20 split. This strategy ensures that evaluation samples are distributed throughout the tool-life trajectory, covering different stages of tool wear. It enables a systematic assessment of the model’s ability to estimate tool wear at different cutting passes within the observed machining process.

For the TC18 D1 reference network, the AVMD-reconstructed cutting force and vibration signals are processed by multiscale convolution to produce 96 feature channels. CBAM-1 recalibrates these features before DenseNet, which produces an 80-channel representation through dense connections and transition compression. Sequence folding then prepares the features for the three-layer BiLSTM. With 128 hidden units per direction, the BiLSTM produces 256 output channels. Following CBAM-2, sequence unfolding and global average pooling yield a 128-dimensional learned representation.

In parallel, 24 statistical features are extracted from each of the three force and two vibration channels, comprising 11 time-domain descriptors, three spectral descriptors, two spectral-peak descriptors, and eight wavelet-packet energy features. These provide 120 features. For the five recorded AE parameters—amplitude, energy, counts, duration, and rise time—the mean, standard deviation, maximum, and minimum are calculated, producing 20 additional features. The resulting 140-dimensional engineered feature vector is concatenated with the 128-dimensional learned representation to form a 268-dimensional input to the regression head.

**Figure 13:**
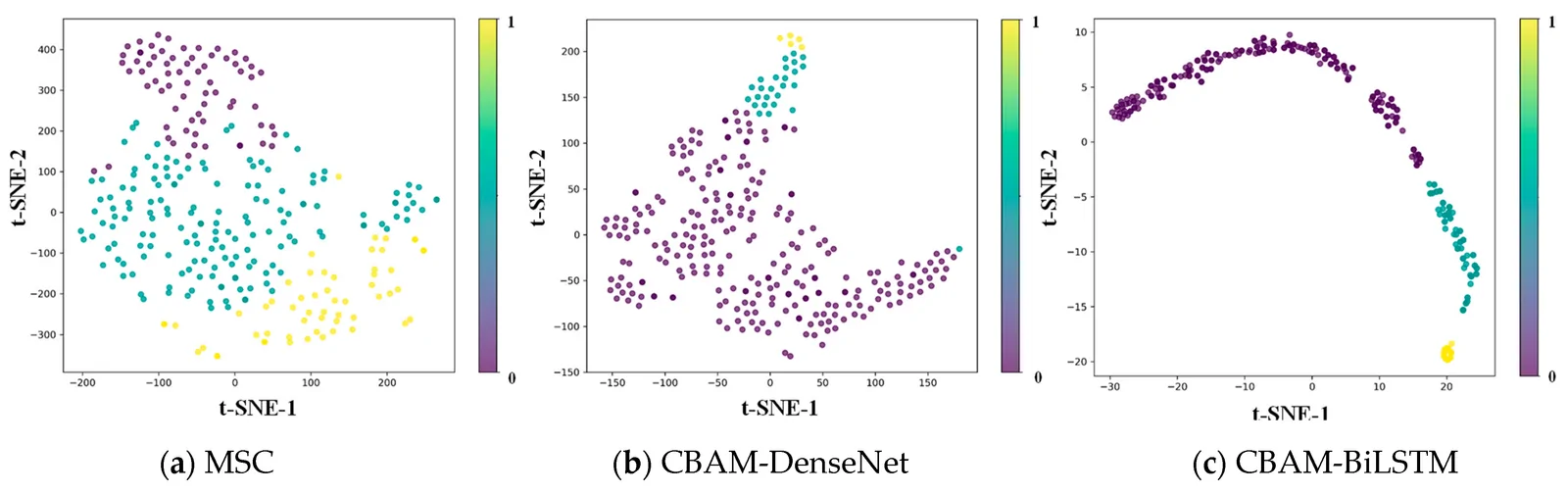
Exploratory t-SNE projections of D1 feature representations: (**a**) MSC output; (**b**) output after CBAM-1 and DenseNet; (**c**) output after BiLSTM, CBAM-2, and sequence unfolding.

Figure 13 provides an exploratory visualization of the D1 feature representations at three processing stages: MSC, CBAM-1 followed by DenseNet, and BiLSTM followed by CBAM-2. Sequence-averaged representations of 96, 80, and 128 dimensions, respectively, are projected into two dimensions using t-SNE. The projections illustrate changes in local sample organization across the feature extraction stages and provide a qualitative view of the resulting representations. Prediction accuracy and the contributions of the two CBAM stages and other feature extraction components are evaluated separately through the prediction comparisons and ablation experiments reported in Section 4.3.

### 4.3. Tool Wear Prediction Results Based on TC18 Milling Data

The TC18 experiments adopted the within-condition data partitioning strategy described in Section 4.2. For each cutting condition, samples were ordered by cutting-pass number, with every fifth sample assigned to the evaluation subset and the remaining samples used for training. The same evaluation samples were used across the compared model configurations within each condition, ensuring consistent evaluation settings. This arrangement allows the prediction performance of different models to be compared across representative stages of the observed tool-life trajectory.

To quantitatively assess the predictive performance of the proposed model, the Root Mean Squared Error (RMSE), Mean Absolute Error (MAE), and the coefficient of determination (R^2^) are employed as evaluation metrics. The lower the RMSE and MAE values, and the closer R^2^ is to 1, the better the predictive performance of the model. The formulas for these evaluation metrics are as follows:

(17) $$RMSE=\sqrt{\frac{1}{N}\sum_{i=1}^{N}{\left(y_{i}-{\hat{y}}_{i}\right)}^{2}}$$

(18) $$MAE=\frac{1}{N}\sum_{i=1}^{N}\left|y_{i}-{\hat{y}}_{i}\right|$$

(19) $$R^{2}=1-\frac{\sum_{i=1}^{N}{\left(y_{i}-{\hat{y}}_{i}\right)}^{2}}{\sum_{i=1}^{N}{\left(y_{i}-\bar{y}\right)}^{2}}$$

In the equation, $y_{i}$ represents the actual wear value of the tool, ${\hat{y}}_{i}$ represents the predicted wear value of the tool, N is the total number of samples in the test set, and $\bar{y}$ denotes the mean of the actual wear values of the tool.

The proposed framework is compared with CNN–BiLSTM, DRSN–BiLSTM, DenseNet–BiGRU, TCN, and Transformer predictors under D1, D2, and D3. TCN and Transformer are included as additional time-series modeling baselines. All prediction metrics are recalculated from the corresponding per-cut prediction files on the evaluation subset of each condition. Table 9 summarizes the recorded results.

The TC18 model comparisons used the same evaluation records within each cutting condition. The proposed framework used five waveform channels together with a 140-dimensional engineered-feature vector, which was concatenated with the learned representation before the regression head. For the proposed framework, waveform inputs, engineered features, and wear targets were standardized using transformations fitted on the training subset and subsequently applied unchanged to the remaining subsets. Predictions were inverse-transformed to micrometres before calculating RMSE, MAE, and R^2^. For configurations evaluated through repeated training with multiple random seeds, each run was initialized independently, and prediction metrics were calculated separately for each run before being averaged across seeds. The comparisons therefore describe the performance of the evaluated model configurations.

For configurations incorporating additional refinement, the final linear layer was optimized using ridge regression with a regularization parameter of 100, followed by fine-tuning with the AdamW optimizer. The learning rate and weight decay were both set to 0.0001, with a batch size of 64 and a maximum of 160 training epochs. A ReduceLROnPlateau scheduler was employed to adaptively adjust the learning rate, reducing it by a factor of 0.5 after 10 epochs without improvement in the monitored loss. Early stopping with a patience of 30 epochs was applied to control the training process.

As shown in Table 9, the proposed framework achieved RMSE values of 12.0554, 7.4098, and 11.1366 μm; MAE values of 6.4693, 4.7217, and 6.7580 μm; and R^2^ values of 0.9399, 0.9470, and 0.9573 under D1, D2, and D3, respectively. The proposed framework outperformed all five comparison models across the three cutting conditions in terms of RMSE, MAE, and R^2^. Compared with the best competing result for each metric, the RMSE reductions were 18.84%, 46.43%, and 6.85%, while the MAE reductions were 12.57%, 39.36%, and 2.87% under D1, D2, and D3, respectively. The corresponding absolute improvements in R^2^ were 0.0311, 0.1316, and 0.0066. Notably, the most pronounced improvements were observed under D2, indicating the effectiveness of the proposed framework in this cutting condition. Although the performance gains were relatively modest under D3, the proposed framework consistently achieved the highest prediction accuracy among the evaluated models.

Controlled ablation experiments were conducted using the full model as the reference configuration. Each variant modified only the designated component or attention configuration, while retaining the same AVMD inputs, engineered-feature branch, data partition, training settings, and checkpoint-selection criterion. Dimension-matching operations were introduced only where required to connect the remaining modules, and these operations were specified for each variant. Each ablation variant was independently initialized and trained using the same set of multiple random seeds as the full model. All other experimental settings were held constant across the seed-specific runs. Results are reported as mean metrics across independently initialized training runs using multiple random seeds.

Under D1, the proposed framework achieved an R^2^ of 0.9399, compared with 0.9320, 0.8630, and 0.9156 when CBAM-1, CBAM-2, and both CBAM modules were removed, respectively. In particular, the complete framework reduced RMSE by 33.77% compared with the variant without CBAM-2, highlighting the contribution of the second attention stage under D1. Under D2, the proposed framework achieved the best overall performance, with an RMSE of 7.4098 μm, an MAE of 4.7217 μm, and an R^2^ of 0.9470. Compared with the variants without CBAM-1 and CBAM-2, its RMSE was reduced by 44.95% and 51.04%, respectively, demonstrating the substantial contributions of both attention stages under this condition. Replacing multiscale convolution with single-scale convolution, replacing DenseNet with a projection module, or removing BiLSTM generally degraded prediction performance. Notably, removing BiLSTM under D3 increased RMSE by 152.52%, from 11.1366 to 28.1216 μm, while reducing R^2^ from 0.9573 to 0.7275. However, removing CBAM-2 under D3 slightly reduced RMSE by 2.07% and improved R^2^ to 0.9590. Overall, these results demonstrate the importance of the principal feature extraction components while indicating that the contributions of individual attention stages vary across cutting conditions.

**Figure 14:**
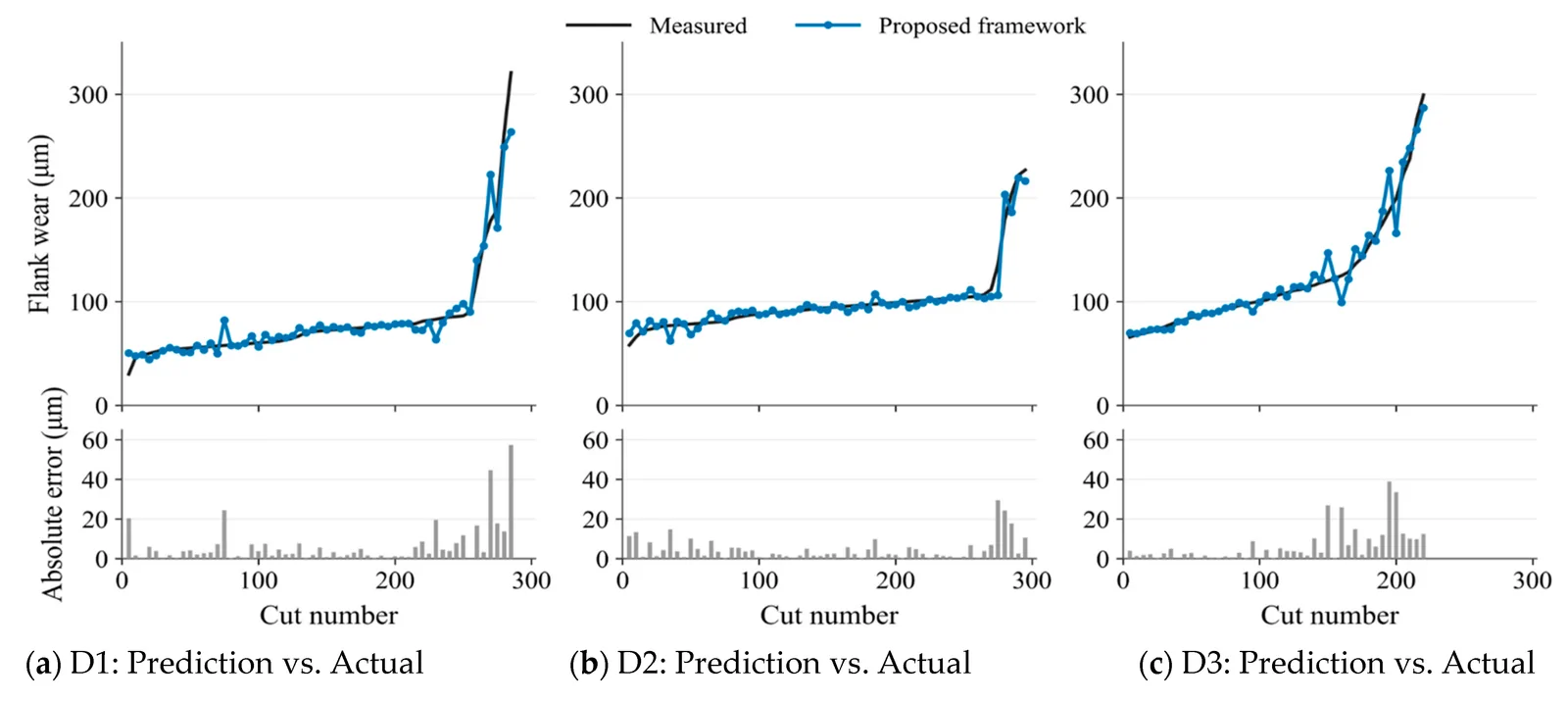
Measured and predicted flank wear (upper panels) and corresponding absolute errors (lower panels) under TC18 conditions (**a**) D1, (**b**) D2, and (**c**) D3.

Figure 14 presents the prediction results under TC18 conditions D1, D2, and D3. The upper panels compare measured and predicted flank wear at the test cutting-pass indices, while the lower panels show the corresponding absolute prediction errors in micrometres. Each error is calculated from the measured–predicted pairs in the displayed seed-specific run. Table 9 reports the means and sample standard deviations of the metrics across multiple random seeds. These plots show how prediction errors vary over the evaluated tool-life trajectories.

**Figure 15:**
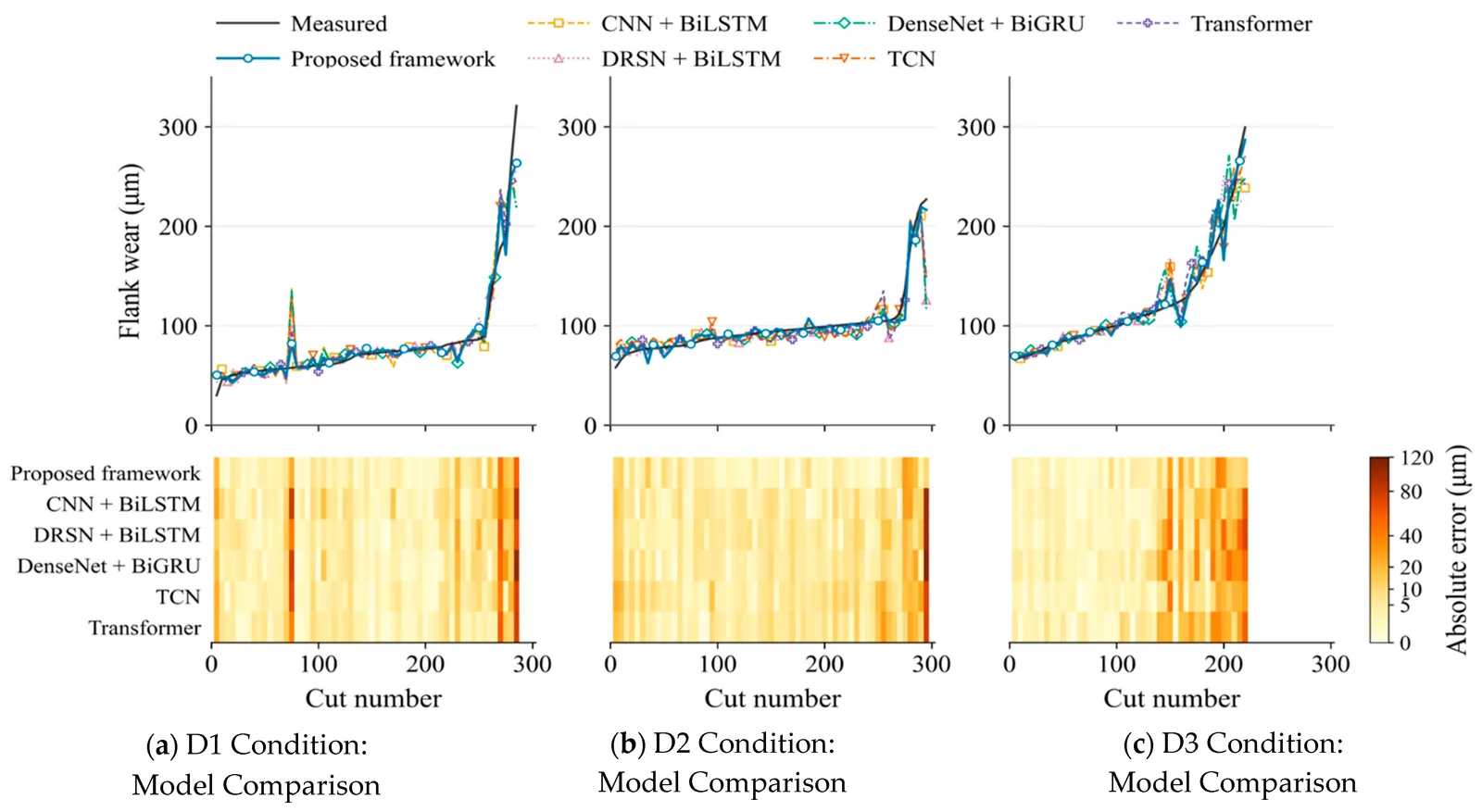
Prediction curves (upper panels) and per-cut absolute-error maps (lower panels) of the proposed framework and five comparison models under TC18 conditions (**a**) D1, (**b**) D2, and (**c**) D3.

Figure 15 compares the proposed framework with CNN–BiLSTM, DRSN–BiLSTM, DenseNet–BiGRU, TCN, and Transformer under the three TC18 conditions. The upper panels show the measured wear and the predictions from each model, with different line styles and markers distinguishing the methods. The lower panels display absolute-error maps, with each row representing one method and each column corresponding to an evaluated cutting pass. Darker colors indicate larger errors. The three error maps share a square-root color scale, with color-bar values expressed in micrometres. All plotted results correspond to Table 9.

**Figure 16:**
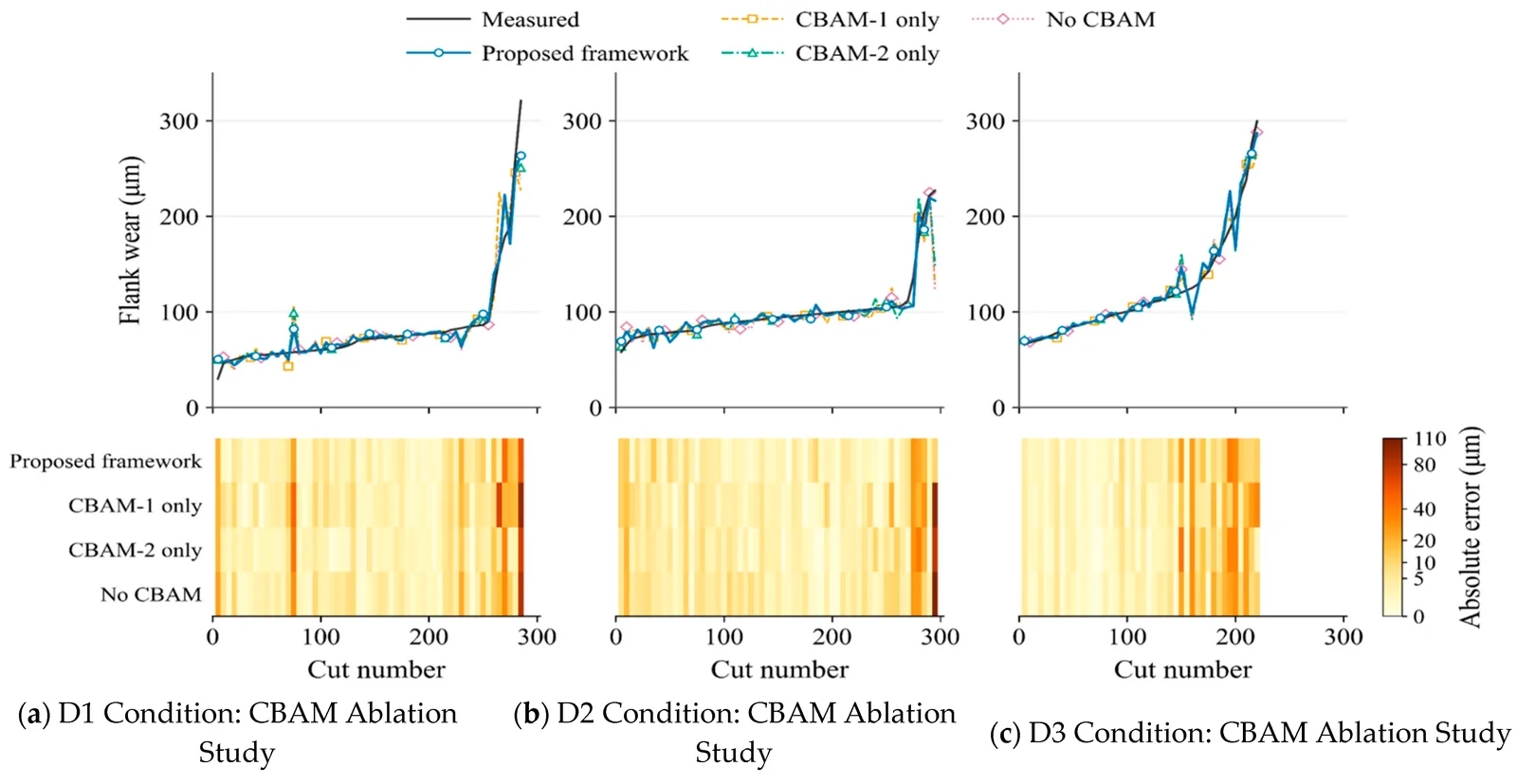
Prediction curves (upper panels) and per-cut absolute-error maps (lower panels) for the four CBAM configurations under TC18 conditions (**a**) D1, (**b**) D2, and (**c**) D3. The error maps share a common color scale for absolute prediction error (µm).

Figure 16 further illustrates the effects of different attention configurations on tool wear prediction under the three TC18 cutting conditions. The two-stage CBAM configuration is compared with three variants: CBAM-1 only, CBAM-2 only, and No CBAM. The prediction curves and corresponding absolute-error distributions provide a visual comparison of the differences among these configurations. Combined with the quantitative results in Table 10, these observations further illustrate the contributions of the two attention stages to prediction performance under different cutting conditions.

Table 11 summarizes the computational cost and GPU forward latency of the evaluated network implementations. The saved D1, D2, and D3 reference models each contain approximately 1.203 million parameters and require 267.037 million matrix multiply–accumulate operations (MACs) per sample, corresponding to approximately 0.5341 GFLOPs under the 2 × MACs convention. Their measured median GPU forward latencies are 5.193, 5.199, and 5.195 ms, respectively, indicating comparable computational requirements across the three cutting conditions. The proposed framework requires fewer matrix MACs than CNN + BiLSTM and DRSN + BiLSTM, although DenseNet + BiGRU, TCN, and Transformer exhibit lower computational requirements. All measurements were conducted using an NVIDIA GeForce RTX 4060 Laptop GPU with a batch size of 1.

### 4.4. Within-Tool and Cross-Tool Evaluation on the PHM 2010 Dataset

The PHM 2010 dataset was used to further evaluate the proposed framework on additional tools under fixed cutting conditions. Two evaluation settings were considered: within-tool prediction and cross-tool evaluation.

The PHM 2010 experiments employed within-tool and cross-tool evaluation strategies. For cross-tool prediction, waveform signals, engineered features, and wear labels were standardized using source-tool training data, and predictions were converted back to micrometres for evaluation. Model training used MSE loss and Adam with an initial learning rate of 0.001 and weight decay of 0.00001. A cosine annealing scheduler adjusted the learning rate to a minimum of 0.000001 over a maximum of 200 epochs. The same multi-seed training and metric-averaging procedure was applied to the PHM 2010 comparisons. Within each evaluation protocol, every comparison model and attention variant was independently initialized and trained using the same set of multiple random seeds. Prediction metrics were calculated separately for each seed-specific run and then averaged across seeds.

For within-tool prediction, every fifth cut of each tool was assigned to the evaluation subset, resulting in 252 training cuts and 63 evaluation cuts for each of C1, C4, and C6. As shown in Table 12, the proposed framework achieved RMSE values of 2.2832, 3.3982, and 3.8524 μm; MAE values of 1.7285, 2.1354, and 2.9030 μm; and R^2^ values of 0.9929, 0.9921, and 0.9907 for C1, C4, and C6, respectively, demonstrating high prediction accuracy within individual tool records.

For cross-tool evaluation, two complete tool records comprising 630 cuts were used as source data, while the remaining tool containing 315 cuts served as the evaluation target. Three source-to-target combinations were considered: C1 + C4 → C6, C1 + C6 → C4, and C4 + C6 → C1. The corresponding RMSE values were 8.0258, 12.2654, and 5.5236 μm; MAE values were 5.9854, 9.0855, and 4.2396 μm; and R^2^ values reached 0.9599, 0.8954, and 0.9591, respectively. Although prediction errors increased compared with the within-tool evaluations, all three cross-tool combinations achieved R^2^ values above 0.895, with two exceeding 0.959. These results indicate that the proposed framework can maintain predictive capability across different tools under fixed cutting conditions, while its prediction performance varies depending on the target tool.

Table 13 compares the within-tool prediction performance of the proposed framework with five baseline models on the PHM 2010 dataset. The proposed framework achieved consistently high prediction accuracy across C1, C4, and C6, with R^2^ values of 0.9929, 0.9921, and 0.9907, respectively. In particular, the proposed framework achieved the best performance on C4, obtaining the lowest RMSE (3.3982 μm) and MAE (2.1354 μm) and the highest R^2^ (0.9921) among all compared models. For C6, the proposed framework achieved the second-best performance across all three evaluation metrics. More importantly, when averaged equally across the three tools, the proposed framework achieved the lowest mean RMSE of 3.1779 μm, the lowest mean MAE of 2.2556 μm, and the highest mean R^2^ of 0.9919. Compared with the strongest competing model for each averaged error metric, the mean RMSE and MAE were reduced by 11.25% and 4.03%, respectively. Although Transformer and TCN achieved slightly higher R^2^ values on C1 and C6, respectively, the proposed framework demonstrated the best overall average prediction performance across the three tools, indicating its ability to maintain accurate and relatively consistent wear estimates under the evaluated within-tool conditions.

Table 14 compares the prediction performance of the proposed framework with five baseline models across three source-to-target tool combinations on the PHM 2010 dataset under fixed cutting conditions. The proposed framework consistently outperformed all five comparison models, achieving the lowest RMSE and MAE and the highest R^2^ in all three evaluation tasks. Specifically, for C1 + C4 → C6, C1 + C6 → C4, and C4 + C6 → C1, the proposed framework achieved RMSE values of 8.0258, 12.2654, and 5.5236 μm; MAE values of 5.9854, 9.0855, and 4.2396 μm; and R^2^ values of 0.9599, 0.8954, and 0.9591, respectively. Compared with the strongest baseline results in each task, the proposed framework consistently improved all three evaluation metrics. These results demonstrate the effectiveness of the proposed framework in maintaining competitive tool wear prediction accuracy across the evaluated tool combinations under fixed cutting conditions.

Table 15 presents the attention ablation results for three cross-tool prediction tasks on the PHM 2010 dataset. The dual-CBAM configuration achieved the lowest RMSE and highest R^2^ across all three tasks, demonstrating the effectiveness of incorporating attention at two distinct feature processing stages. Specifically, the dual-CBAM configuration achieved R^2^ values of 0.9599, 0.8954, and 0.9591 for C1 + C4 → C6, C1 + C6 → C4, and C4 + C6 → C1, respectively. Overall, these results support the effectiveness of the two-stage attention mechanism for improving prediction accuracy across the evaluated tool combinations.

**Figure 17:**
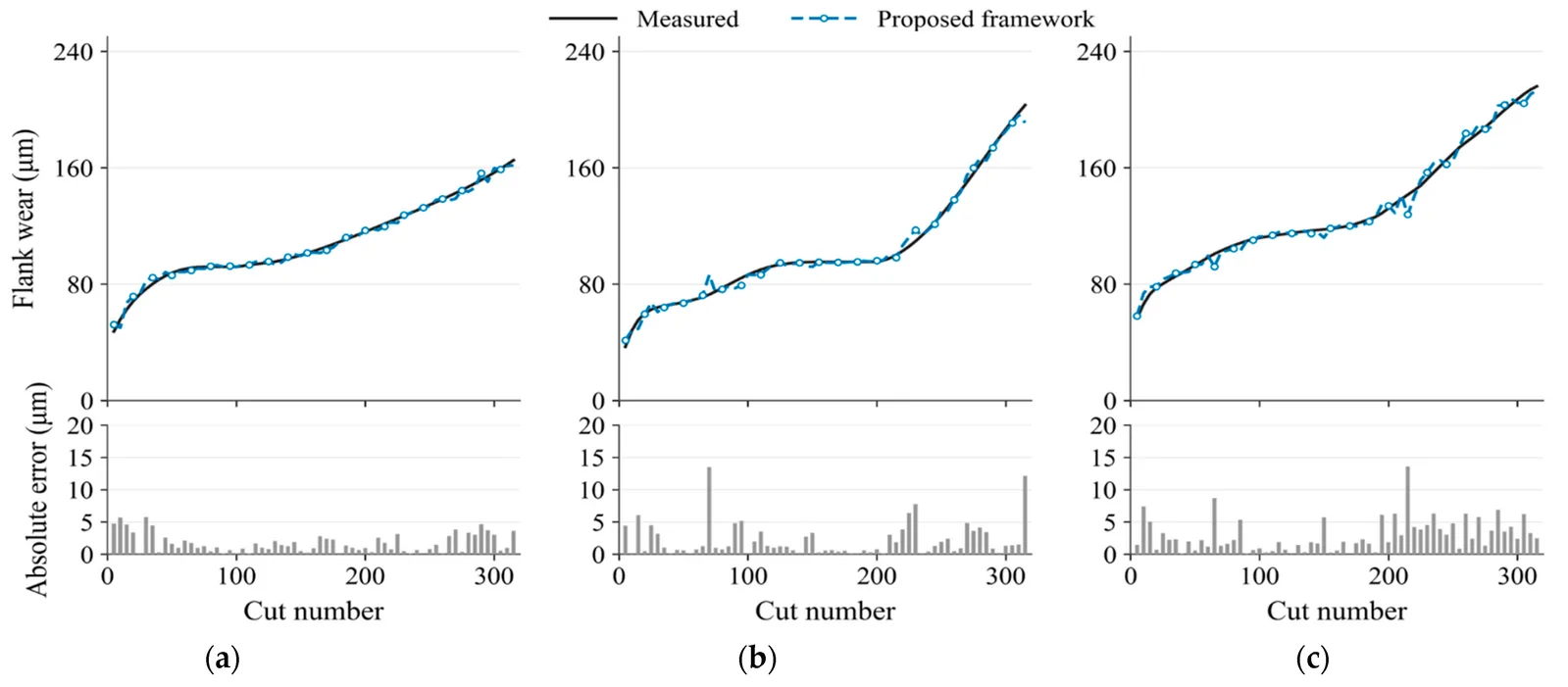
Within-tool prediction results for PHM 2010 tools C1, C4, and C6. (**a**) C1: Prediction vs. Actual. (**b**) C4: Prediction vs. Actual. (**c**) C6: Prediction vs. Actual.

Figure 17 presents the within-tool prediction results for C1, C4, and C6. The upper panels compare measured and predicted flank wear at the 63 evaluation cutting passes of each tool, and the lower panels show the corresponding absolute errors. All curves and errors are calculated from the prediction files used in Table 12.

**Figure 18:**
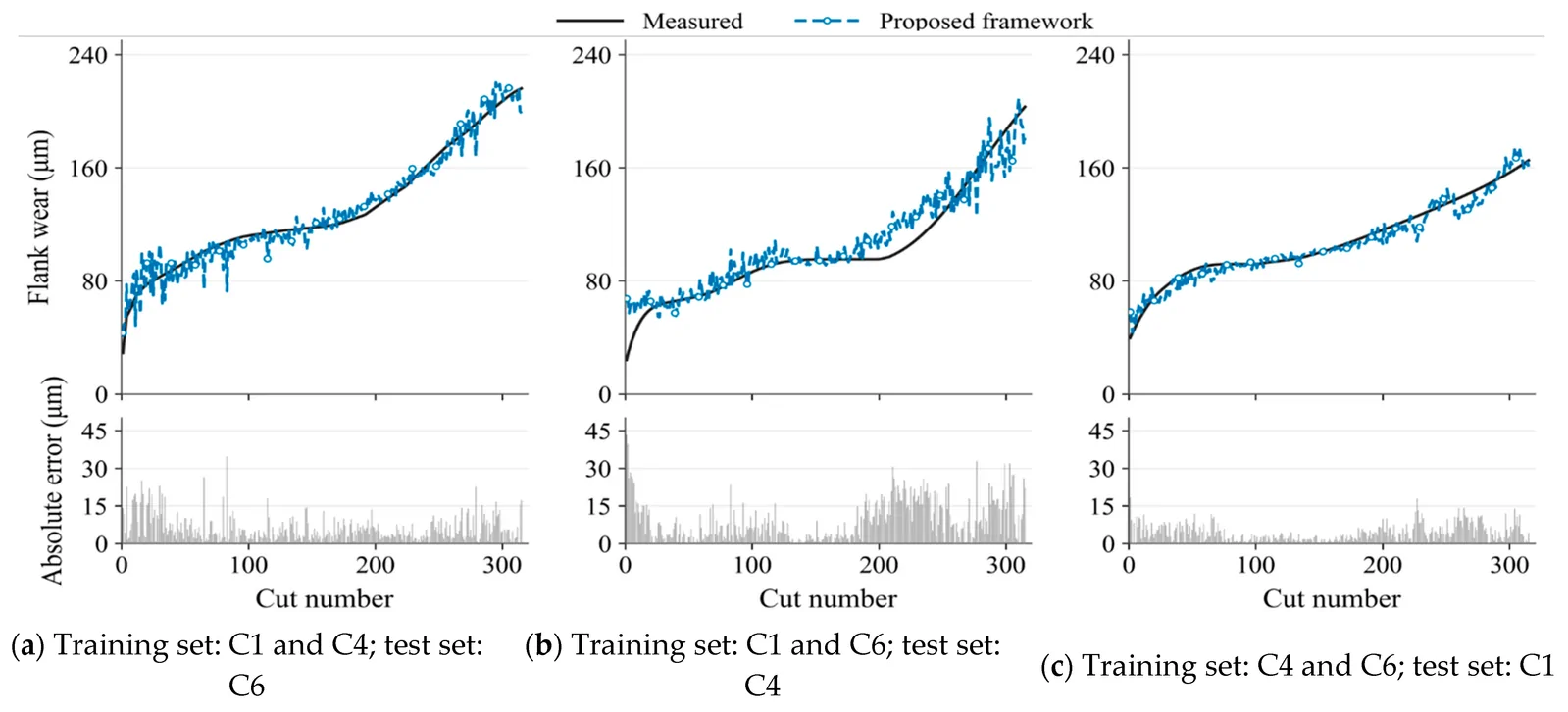
Recorded prediction curves (upper panels) and absolute errors (lower panels) for (**a**) C1 + C4 → C6, (**b**) C1 + C6 → C4, and (**c**) C4 + C6 → C1.

Figure 18 presents the cross-tool prediction results for three source-to-target combinations: C1 + C4 → C6, C1 + C6 → C4, and C4 + C6 → C1. The upper panels compare the measured and predicted tool wear values across 315 evaluation cuts for each target tool, while the lower panels illustrate the corresponding absolute prediction errors. The predicted wear trajectories generally follow the measured trends across all three tasks, demonstrating the predictive capability of the proposed framework for different tool combinations under fixed cutting conditions. These visual results further support the quantitative evaluations presented in Table 12.

Overall, the PHM 2010 experimental results demonstrate the effectiveness of the proposed framework in tool wear prediction under fixed cutting conditions. The framework achieved the best average prediction performance across the three within-tool evaluations and consistently outperformed all five baseline models in the three cross-tool comparison tasks. Furthermore, the attention ablation results confirmed the advantages of the two-stage CBAM configuration in improving prediction accuracy. These findings highlight the effectiveness of integrating multiscale feature extraction, dense feature reuse, bidirectional temporal modeling, and two-stage attention for accurate and reliable tool wear prediction.

### 4.5. SHAP-Based Interpretability Analysis

SHAP is a game-theoretic approach for explaining model predictions through additive feature attributions [47]. For the regression-head input x, the prediction can be expressed as

(20) $$h(x)=\phi_{0}+\sum_{j=1}^{M}\phi_{j}(x)$$

where h denotes the trained regression head with its output converted to the original wear scale, M is the number of input coordinates, $\phi_{0}$ is the background reference prediction, and $\phi_{j}\left(x\right)$ is the attribution of coordinate j. Positive and negative attributions indicate increases and decreases in the predicted wear relative to the background reference, respectively.

The attribution of coordinate j is defined as

(21) $$\phi_{j}(x)=\sum_{S\subseteq F\setminus \{j\}}\frac{|S|!(M-|S|-1)!}{M!}\left[v_{x}(S\cup \{j\})-v_{x}(S)\right]$$

where $F=\left\{1,\ldots ,M\right\}$, S is a subset excluding coordinate j, and $\left|S\right|$ is its cardinality. The coalition value $v_{x}\left(S\right)$ is the expected regression-head output when coordinates in S retain their observed values and the remaining coordinates are replaced by values from training-background vectors.

To further investigate the interpretability of the proposed framework, SHAP analysis was conducted on the trained regression heads of the TC18 prediction models. Each model incorporates a 268-dimensional feature representation, consisting of 128 learned latent features and 140 engineered features. SHAP values were calculated to quantify the contributions of individual features to the predicted tool wear values, providing insights into their relative importance and influence on model predictions. This analysis facilitates a better understanding of how the learned representations and engineered statistical features contribute to tool wear estimation.

For each cutting condition, 16 training samples were selected as the background set, and 12 evaluation samples distributed across the recorded cutting-pass sequence were selected for explanation. A Monte Carlo interventional permutation Shapley estimator was employed, using 32 random permutations per explained sample and a fixed random seed of 42. The resulting SHAP values were expressed in micrometres (μm), consistent with the output units of the regression model.

**Figure 19:**
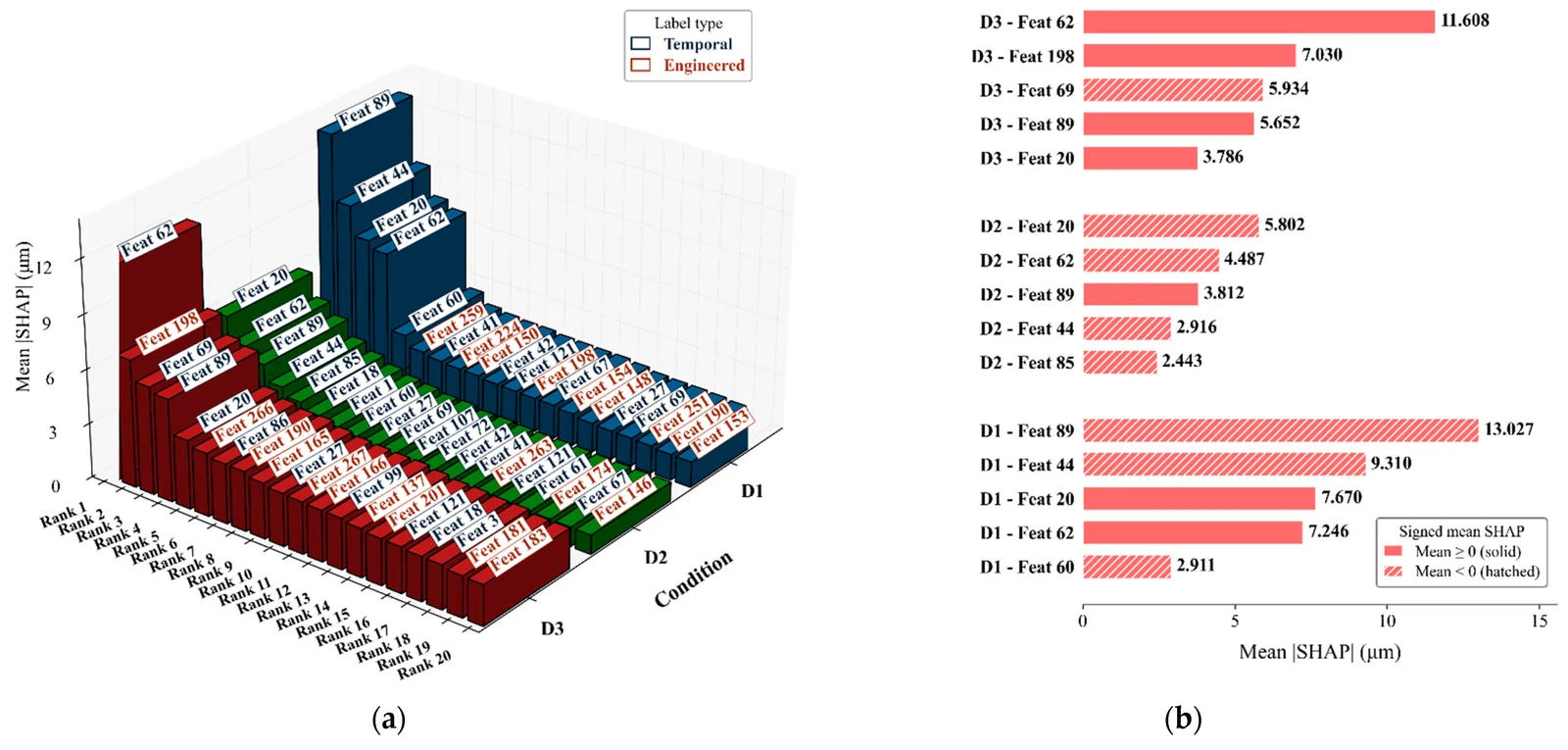
SHAP-based feature importance analysis under three TC18 cutting conditions. (**a**) Top 20 Features Ranked by Mean |SHAP|. (**b**) Top Five Features and SHAP Attribution Directions.

Figure 19 presents the SHAP-based feature importance analysis under the three TC18 cutting conditions. Feature importance was quantified using the mean absolute SHAP values across the 12 explained samples for each condition. The results show that both automatically learned temporal features and engineered statistical features contribute to tool wear prediction, with their relative importance varying across cutting conditions. Specifically, Temporal feature 89 (Feat 89) exhibited the highest importance under D1, with a mean absolute SHAP value of 13.027 μm, while Temporal feature 20 (Feat 20) ranked first under D2, reaching 5.802 μm. Under D3, Temporal feature 62 (Feat 62) showed the largest contribution of 11.608 μm, followed by the standard deviation of the *F_z_* cutting-force signal (Feat 198), with a mean absolute SHAP value of 7.030 μm. The engineered features include statistical descriptors of signal amplitude, variability, frequency characteristics, and wavelet-packet energy. Among these, the standard deviation of *F_z_* provides a physically interpretable measure of cutting-force fluctuations, offering additional insight into the signal characteristics associated with the model predictions. These findings highlight the complementary contributions of learned temporal representations and engineered signal features to tool wear estimation.

The signed SHAP values further illustrate the direction and magnitude of individual feature contributions to tool wear predictions. Positive and negative SHAP values indicate whether individual features increase or decrease the predicted wear relative to the model baseline, while variations across samples reflect differences in feature contributions. By combining global feature importance with sample-level attribution analysis, SHAP provides complementary insights into the prediction behavior of the proposed framework. Furthermore, the ablation experiments presented in Section 4.3 evaluate the contributions of the feature extraction and attention modules to prediction performance, complementing the SHAP-based interpretability analysis.

## 5. Conclusions

This study developed a tool wear prediction framework combining adaptive variational mode decomposition (AVMD), multi-scale convolution, and two-stage attention for multi-sensor milling data. Channel-specific decomposition is used for signal preprocessing, while convolutional and recurrent components extract complementary feature representations. The framework was evaluated using the TC18 variable-condition milling dataset and the PHM 2010 dataset. The main conclusions are as follows:

(1) A data-informed AVMD parameter optimization strategy was developed to adapt decomposition parameters to individual sensor channels. By integrating spectral-feature-guided candidate generation with decomposition-based evaluation criteria, the proposed strategy enables efficient and repeatable parameter selection. In the supplementary experiments, three independent searches produced consistent parameter configurations, with average calibration times of 88.48 ± 1.21 s and 168.03 ± 3.73 s for the TC18 and PHM 2010 benchmarks, respectively. Compared with the alternative optimization methods, AVMD achieved lower computational costs, demonstrating its efficiency and parameter-selection stability under the evaluated settings.
(2) A two-stage attention approach was introduced to refine feature representations at different processing stages. Multi-scale convolution captures local signal patterns, while the convolutional and recurrent components support feature fusion and temporal modeling. The ablation experiments provide empirical guidance for selecting attention placement and feature extraction configurations for different tasks. The t-SNE visualizations illustrate the resulting feature representations, and SHAP analysis provides complementary insight into the contributions of learned temporal features and engineered statistical features across the examined predictors.
(3) The reported TC18 configurations achieved R^2^ values of 0.9399, 0.9470, and 0.9573 under D1, D2, and D3, respectively. On PHM 2010, within-tool prediction achieved R^2^ values of 0.9929, 0.9921, and 0.9907 for C1, C4, and C6, respectively. For the C1 + C4 → C6 and C4 + C6 → C1 prediction tasks, the corresponding R^2^ values were 0.9599 and 0.9591. These results demonstrate the framework’s predictive capability across the evaluated milling datasets and support its potential for multi-sensor tool wear monitoring.

Future work will focus on model compression, embedded deployment, and end-to-end processing efficiency. Dedicated experiments will assess robustness under controlled noise levels and end-to-end online performance, which were not established in the present study. Further experiments involving additional tools, materials, and operating conditions will extend the evaluation of the framework. Transfer learning, domain adaptation, and the integration of machining knowledge will also be explored to improve adaptability and interpretability for industrial applications.

## Figures and Tables

**Table 1:** Milling Parameters for TC18 Under Three Operating Conditions.

No.	Cutting Parameters			
	Cutting Speed (m/min)	Feed per Tooth(mm/r)	Cutting Width (mm)	Cutting Depth (mm)
Group 1 (D1)	100.48	0.06	3	0.1
Group 2 (D2)	75.36	0.05	5	0.1
Group 3 (D3)	75.36	0.04	3	0.4

**Table 2:** Milling parameters for the PHM 2010 dataset.

No.	Cutting Parameters			
	Spindle Speed (r/min)	Feed Rate (mm/min)	Cutting Width (mm)	Cutting Depth (mm)
C1, C2, C3, C4, C5, C6	10,400	1555	0.125	0.2

**Table 3:** Archived channel-specific AVMD parameters for the TC18 dataset.

Condition	Fx	Fy	Fz	Vx	Vy
D1	(4, 1867, 0.183)	(3, 2471, 0.189)	(3, 1694, 0.193)	(4, 1606, 0.779)	(4, 636, 0.796)
D2	(6, 605, 0.517)	(2, 1739, 0.230)	(2, 1694, 0.202)	(4, 967, 0.779)	(4, 605, 0.783)
D3	(4, 605, 0.355)	(2, 4027, 0.212)	(2, 2471, 0.218)	(3, 967, 0.782)	(4, 605, 0.788)

**Table 4:** Summed channel calibration times under the supplementary search configurations.

Method	TC18 D1 (s)	PHM 2010 C6 (s)
AVMD	88.48 ± 1.21	168.03 ± 3.73
PSO-VMD	154.70 ± 7.96	232.30 ± 56.83
DE-VMD	190.32 ± 13.17	252.43 ± 33.65
Envelope-entropy VMD	359.91 ± 322.68	267.18 ± 34.93

Values are mean ± sample SD from three complete searches (*n* = 3). VMD calls per search were 822 for all methods on TC18 D1, and 2370 for AVMD versus 2358 for the other methods on PHM 2010. Benchmarks used an Intel Core i5-13500HX CPU and RTX 4060 Laptop GPU, with Python 3.9.25, PyTorch 2.5.1, and CUDA 12.4. Settings comprised one search worker, four PyTorch CPU threads, and GPU batches of up to 32 decompositions. Calibration used three TC18 and six PHM training records per channel. Methods were timed in separate batches, excluding data loading, GPU warm-up, and downstream reconstruction or prediction.

**Table 5:** Comparison of signal reconstruction performance using different VMD methods under TC18 cutting conditions.

Method	D1		D2		D3	
	RMSE	SNR (dB)	RMSE	SNR (dB)	RMSE	SNR (dB)
AVMD	0.02692	23.41	0.00371	44.42	0.01004	56.33
Fixed VMD	0.02357	25.33	0.02015	24.82	0.01984	25.41
PSO-VMD	0.02632	25.19	0.01214	30.15	0.01913	27.05
DE-VMD	0.02915	22.83	0.00855	44.79	0.01005	56.33
Envelope-entropy VMD	0.02622	23.51	0.01616	28.09	0.01443	41.43

**Table 6:** Parameter-selection stability of different VMD optimization methods.

Calibration Case	Method	K	α	τ
TC18 D1	AVMD	4.00 ± 0.00	1867.00 ± 0.00	0.1830 ± 0.000
TC18 D1	PSO-VMD	5.00 ± 3.00	4215.51 ± 4689.41	0.1773 ± 0.0485
TC18 D1	DE-VMD	3.00 ± 1.73	1960.38 ± 1819.55	0.1909 ± 0.0414
TC18 D1	Envelope-entropy VMD	6.33 ± 1.15	849.42 ± 1026.82	0.0622 ± 0.1034
PHM 2010 C6	AVMD	3.00 ± 0.00	647.72 ± 0.00	0.2500 ± 0.0000
PHM 2010 C6	PSO-VMD	3.00 ± 0.00	1420.72 ± 426.71	0.1391 ± 0.0747
PHM 2010 C6	DE-VMD	4.67 ± 2.89	1760.54 ± 2266.57	0.1526 ± 0.0416
PHM 2010 C6	Envelope-entropy VMD	6.67 ± 1.53	2946.33 ± 2756.70	0.0692 ± 0.1199

**Table 7:** Effect of signal preprocessing on downstream tool wear prediction.

Preprocessing Method	D1			D2			D3		
	RMSE (μm)	MAE (μm)	R^2^	RMSE (μm)	MAE (μm)	R^2^	RMSE (μm)	MAE (μm)	R^2^
Raw signals	12.9088	6.0359	0.9311	16.1554	8.2216	0.7481	9.8549	6.0557	0.9665
Fixed VMD	12.7149	6.2523	0.9332	14.8099	8.2856	0.7883	11.6814	6.8197	0.9530
PSO-VMD	12.4920	5.6544	0.9355	15.1193	7.5532	0.7794	10.9502	6.3649	0.9587
DE-VMD	11.8864	5.7705	0.9416	13.5849	7.9087	0.8219	10.9131	6.4557	0.9590
AVMD (proposed method)	12.0554	6.4693	0.9399	7.4098	4.7217	0.9470	11.1366	6.7580	0.9573

**Table 8:** Architecture and base-stage training settings of the D1 reference network.

Module	Settings	Output Shape
Input	Five waveform channels; 5000 samples per cut	5000 × 5
Multiscale convolution	Three branches, 32 channels each; kernel = 15; dilation = 1, 3, 5; length alignment and average pooling	1250 × 96
CBAM-1	Before DenseNet; reduction ratio = 16; spatial kernel = 7	1250 × 96
Stacked DenseNet	Three dense blocks; growth rate = 32; bottleneck width = 128; two transitions with channel compression and pooling	312 × 80
Sequence folding	Factor = 2	156 × 160
Deep BiLSTM	Three bidirectional layers; 128 units per direction; inter-layer dropout = 0.3	156 × 256
CBAM-2	After BiLSTM; reduction ratio = 16; spatial kernel = 7	156 × 256
Sequence unfolding	Factor = 2	312 × 128
Global average pooling	Temporal pooling followed by dropout = 0.3	128
Feature concatenation	128 learned features + 140 engineered sensor features	268
Regression head	268 → 64 → 32 → 1; ReLU after the first two linear layers; dropout = 0.3 after the first ReLU	1
Base optimization	AdamW; initial learning rate = 0.002; weight decay = 0.0001; batch size = 64; maximum epochs = 200; early-stopping patience = 40	—

**Table 9:** Comparison of tool wear prediction models on the TC18 dataset.

Model/Configuration	D1			D2			D3		
	RMSE (μm)	MAE (μm)	R^2^	RMSE (μm)	MAE (μm)	R^2^	RMSE (μm)	MAE (μm)	R^2^
Proposed framework	12.0554	6.4693	0.9399	7.4098	4.7217	0.9470	11.1366	6.7580	0.9573
CNN + BiLSTM	18.8734	9.7324	0.8527	15.7266	7.9607	0.7613	14.4474	8.3050	0.9281
DRSN + BiLSTM	14.8535	7.7523	0.9088	15.2117	7.8537	0.7767	20.1008	11.0626	0.8608
DenseNet + BiGRU	19.7538	8.7971	0.8387	16.2945	7.7859	0.7438	18.2312	11.5744	0.8855
TCN	16.0699	7.8210	0.8932	14.0955	9.3094	0.8083	11.9556	6.9575	0.9507
Transformer	15.0697	7.3991	0.9061	13.8317	8.3777	0.8154	16.2158	10.5830	0.9094

**Table 10:** Controlled ablation results for attention and feature extraction components on TC18.

Model/Configuration	D1			D2			D3		
	RMSE (μm)	MAE (μm)	R^2^	RMSE (μm)	MAE (μm)	R^2^	RMSE (μm)	MAE (μm)	R^2^
Proposed framework	12.0554	6.4693	0.9399	7.4098	4.7217	0.9470	11.1366	6.7580	0.9573
Without CBAM-1	12.8254	5.8266	0.9320	13.4610	6.4538	0.8251	12.8276	7.0011	0.9433
Without CBAM-2	18.2022	8.7613	0.8630	15.1356	6.8373	0.7789	10.9061	6.4705	0.9590
Without both CBAMs	14.2900	7.3819	0.9156	15.4712	7.1789	0.7690	11.4385	7.0368	0.9549
Single-scale convolution	16.2233	8.6935	0.8912	14.9129	7.6153	0.7854	11.7462	7.0249	0.9524
DenseNet replaced by projection	15.9379	8.3193	0.8950	13.9267	8.2894	0.8128	16.4692	9.0582	0.9065
Without BiLSTM	19.2204	9.0596	0.8473	14.0261	7.2029	0.8101	28.1216	14.8823	0.7275

**Table 11:** Computational cost and GPU forward latency of TC18 network implementations.

Model/Configuration	Parameters (M)	Matrix MACs (M)	2 × MACs (GFLOPs)	GPU Median (ms)
TC18 D1 reference	1.202729	267.037	0.5341	5.193
TC18 D2 reference	1.202729	267.037	0.5341	5.199
TC18 D3 reference	1.202729	267.037	0.5341	5.195
CNN + BiLSTM	1.112641	729.678	1.4594	9.627
DRSN + BiLSTM	1.176333	806.480	1.6130	10.977
DenseNet + BiGRU	0.921105	224.826	0.4497	3.233
TCN	0.185089	108.273	0.2165	4.586
Transformer	0.249601	128.082	0.2562	1.127

Benchmarked in separate batches. GPU latency was measured on an RTX 4060 Laptop GPU with batch size 1, 20 warm-up passes, and 100 timed runs. GFLOPs ≈ 2 × MACs; latency represents network forward computation only.

**Table 12:** PHM 2010 prediction results under within-tool and cross-tool protocols.

Evaluation	Training/Source Data	Evaluation Data	RMSE (μm)	MAE (μm)	R^2^
Within-tool	C1: 252 cuts	C1: 63 cuts	2.2832	1.7285	0.9929
Within-tool	C4: 252 cuts	C4: 63 cuts	3.3982	2.1354	0.9921
Within-tool	C6: 252 cuts	C6: 63 cuts	3.8524	2.9030	0.9907
Cross-tool	C1 + C4: 630 cuts	C6: 315 cuts	8.0258	5.9854	0.9599
Cross-tool	C1 + C6: 630 cuts	C4: 315 cuts	12.2654	9.0855	0.8954
Cross-tool	C4 + C6: 630 cuts	C1: 315 cuts	5.5236	4.2396	0.9591

Within-tool evaluations used 252 training cuts and 63 evaluation cuts per tool, with every fifth cut assigned to the evaluation subset. Cross-tool evaluations used 630 source cuts and 315 evaluation cuts for each source-to-target combination.

**Table 13:** Within-tool prediction comparisons on PHM 2010.

Model	C1			C4			C6		
	RMSE (μm)	MAE (μm)	R^2^	RMSE (μm)	MAE (μm)	R^2^	RMSE (μm)	MAE (μm)	R^2^
Proposed framework	2.2832	1.7285	0.9929	3.3982	2.1354	0.9921	3.8524	2.9030	0.9907
CNN + BiLSTM	2.2249	1.7501	0.9933	3.9788	2.7772	0.9892	4.6250	3.3568	0.9865
DRSN + BiLSTM	2.3312	1.6390	0.9926	3.4371	2.2707	0.9919	5.1350	3.8183	0.9834
DenseNet + BiGRU	2.1999	1.4569	0.9934	3.8682	2.4678	0.9898	4.6739	3.4141	0.9862
TCN	2.3739	1.6106	0.9923	5.2490	3.1643	0.9812	3.2869	2.2759	0.9932
Transformer	1.9209	1.4064	0.9950	5.5352	3.5611	0.9791	4.7824	3.0689	0.9856

**Table 14:** Comparison of cross-tool prediction performance on the PHM 2010 dataset.

Model	C1 + C4 → C6			C1 + C6 → C4			C4 + C6 → C1		
	RMSE (μm)	MAE (μm)	R^2^	RMSE (μm)	MAE (μm)	R^2^	RMSE (μm)	MAE (μm)	R^2^
Proposed framework	8.0258	5.9854	0.9599	12.2654	9.0855	0.8954	5.5236	4.2396	0.9591
CNN + BiLSTM	9.8194	7.4755	0.9400	16.2834	12.0878	0.8157	8.0912	6.5486	0.9122
DRSN + BiLSTM	8.3994	6.2787	0.9561	20.3832	15.5089	0.7112	7.8518	6.5842	0.9173
DenseNet + BiGRU	14.7366	11.4819	0.8648	16.9652	11.8726	0.7999	5.8373	4.7276	0.9543
TCN	14.7575	10.8844	0.8645	25.7900	18.7404	0.5376	6.5451	5.2589	0.9425
Transformer	8.2704	6.4900	0.9574	12.7505	9.1766	0.8870	6.1907	4.9926	0.9486

**Table 15:** Attention-configuration comparisons for PHM 2010 tool combinations.

Attention Configuration	C1 + C4 → C6			C1 + C6 → C4			C4 + C6 → C1		
	RMSE (μm)	MAE (μm)	R^2^	RMSE (μm)	MAE (μm)	R^2^	RMSE (μm)	MAE (μm)	R^2^
Dual CBAM	8.0258	5.9854	0.9599	12.2654	9.0855	0.8954	5.5236	4.2396	0.9591
CBAM-1 only	8.9569	6.5623	0.9501	15.0066	11.9065	0.8434	6.5322	5.1946	0.9428
CBAM-2 only	8.6889	6.4096	0.9530	12.4806	9.7196	0.8917	7.2372	5.8138	0.9298
No CBAM	8.1383	5.7992	0.9588	15.3481	12.2082	0.8362	7.1682	5.8227	0.9311

## Data Availability

The TC18 milling dataset is not publicly available because it forms part of an ongoing research project. Requests to access the dataset should be directed to the corresponding author. The PHM 2010 dataset analyzed in this study is publicly available from the PHM Society Data Challenge repository at https://phmsociety.org/phm_competition/2010-phm-society-conference-data-challenge/ (accessed on 17 October 2026).
